# Biocontrol and plant growth promoting traits of two avocado rhizobacteria are orchestrated by the emission of diffusible and volatile compounds

**DOI:** 10.3389/fmicb.2023.1152597

**Published:** 2023-05-03

**Authors:** Elvis M. Cortazar-Murillo, Alfonso Méndez-Bravo, Juan L. Monribot-Villanueva, Edith Garay-Serrano, Ana L. Kiel-Martínez, Mónica Ramírez-Vázquez, Edgar Guevara-Avendaño, Alejandro Méndez-Bravo, José A. Guerrero-Analco, Frédérique Reverchon

**Affiliations:** ^1^Red de Estudios Moleculares Avanzados, Instituto de Ecología, A.C., Xalapa, Veracruz, Mexico; ^2^CONACyT – Escuela Nacional de Estudios Superiores, Unidad Morelia, Laboratorio Nacional de Análisis y Síntesis Ecológica, Universidad Nacional Autónoma de México, Morelia, Michoacán, Mexico; ^3^CONACyT – Red de Diversidad Biológica del Occidente Mexicano, Centro Regional del Bajío, Instituto de Ecología, A.C., Pátzcuaro, Michoacán, Mexico; ^4^Facultad de Medicina, Universidad Nacional Autónoma de México, Ciudad de México, Mexico; ^5^Escuela Nacional de Estudios Superiores Unidad Morelia, Laboratorio Nacional de Análisis y Síntesis Ecológica, Universidad Nacional Autónoma de México, Morelia, Mexico; ^6^Red de Diversidad Biológica del Occidente Mexicano, Centro Regional del Bajío, Instituto de Ecología, A.C., Pátzcuaro, Michoacán, Mexico

**Keywords:** antifungal polyketides, auxin signaling, *Bacillus*, *Fusarium* spp., *Phytophthora cinnamomi*

## Abstract

Avocado (*Persea americana* Mill.) is a tree crop of great social and economic importance. However, the crop productivity is hindered by fast-spreading diseases, which calls for the search of new biocontrol alternatives to mitigate the impact of avocado phytopathogens. Our objectives were to evaluate the antimicrobial activity of diffusible and volatile organic compounds (VOCs) produced by two avocado rhizobacteria (*Bacillus* A8a and HA) against phytopathogens *Fusarium solani*, *Fusarium kuroshium*, and *Phytophthora cinnamomi*, and assess their plant growth promoting effect in *Arabidopsis thaliana*. We found that, *in vitro*, VOCs emitted by both bacterial strains inhibited mycelial growth of the tested pathogens by at least 20%. Identification of bacterial VOCs by gas chromatography coupled to mass spectrometry (GC–MS) showed a predominance of ketones, alcohols and nitrogenous compounds, previously reported for their antimicrobial activity. Bacterial organic extracts obtained with ethyl acetate significantly reduced mycelial growth of *F. solani*, *F. kuroshium*, and *P. cinnamomi*, the highest inhibition being displayed by those from strain A8a (32, 77, and 100% inhibition, respectively). Tentative identifications carried out by liquid chromatography coupled to accurate mass spectrometry of diffusible metabolites in the bacterial extracts, evidenced the presence of some polyketides such as macrolactins and difficidin, hybrid peptides including bacillaene, and non-ribosomal peptides such as bacilysin, which have also been described in *Bacillus* spp. for antimicrobial activities. The plant growth regulator indole-3-acetic acid was also identified in the bacterial extracts. *In vitro* assays showed that VOCs from strain HA and diffusible compounds from strain A8a modified root development and increased fresh weight of *A. thaliana*. These compounds differentially activated several hormonal signaling pathways involved in development and defense responses in *A. thaliana*, such as auxin, jasmonic acid (JA) and salicylic acid (SA); genetic analyses suggested that developmental stimulation of the root system architecture by strain A8a was mediated by the auxin signaling pathway. Furthermore, both strains were able to enhance plant growth and decreased the symptoms of Fusarium wilt in *A. thaliana* when soil-inoculated. Collectively, our results evidence the potential of these two rhizobacterial strains and their metabolites as biocontrol agents of avocado pathogens and as biofertilizers.

## 1. Introduction

Soil microorganisms, in particular those inhabiting the rhizosphere, greatly contribute to plant health and productivity by mediating their nutrient uptake, enhancing their tolerance to biotic and abiotic stressors, and by producing phytohormones involved in plant growth promotion (Bulgarelli et al., 2013; Philippot et al., 2013; Bejarano-Bolívar et al., 2021). As a consequence, plant growth promoting rhizobacteria (PGPR) have been widely explored for their potential application as biofertilizers or biological control agents of phytopathogens (Jiao X. et al., 2021; Santoyo, 2022; Méndez-Bravo et al., 2023), and represent a promising sustainable alternative to the use of agrochemicals. However, a deeper understanding of the mechanisms underlying plant growth promoting or antimicrobial activities displayed by PGPR is necessary to harness their full potential.

Recent studies have shed a light on bacterial bioactive compounds and their possible use for sustainable food production. Plant growth promotion by the indole acetic acid (IAA) hormone or by volatile organic compounds (VOCs) like dimethyl disulfide (DMDS), isoamyl acetate, or 3-methyl-1-butanol, has been demonstrated in model and non-model hosts such as *Arabidopsis thaliana, Nicotiana attenuata* or *Agave* spp. (Meldau et al., 2013; Méndez-Bravo et al., 2018; Camarena-Pozos et al., 2019). Antimicrobial peptides or VOCs such as aliphatic ketones and sulfur compounds are able to induce plant defense responses and/or directly antagonize fungal pathogens (Guevara-Avendaño et al., 2019; Nazari and Smith, 2020; Praveena et al., 2022). In this context, PGPR belonging to the *Bacillus* genus are particularly interesting, due to their ubiquity, ability to form endospores and tolerate harsh environmental conditions, their capacity to colonize roots, promote plant growth and development, induce systemic resistance in their hosts, and produce a wide range of specialized metabolites with antimicrobial activities (Dimkić et al., 2017; Guevara-Avendaño et al., 2018; Khatoon et al., 2022; Luo et al., 2022). Such metabolites include diffusible compounds such as cyclic lipopeptides or polyketides, which have been largely described for their antimicrobial activity against a vast array of plant pathogens (Gong et al., 2015; Caulier et al., 2019; Guevara-Avendaño et al., 2020). Moreover, antimicrobial VOCs emitted by *Bacillus* spp., such as 2-decanone, 2,3,5-trimethylpyrazine or 2-methylbutanoic acid, exhibited antagonistic activity against phytopathogens such as *Alternaria alternata*, *Fusarium solani*, *Fusarium kuroshium*, and *Phytophthora cinnamomi* (Asari et al., 2016; Méndez-Bravo et al., 2018; Wang et al., 2022). Previous findings have emphasized the huge potential of *Bacillus* spp. as biofertilizers or biocontrol agents, although strain-specific plant growth promoting traits and antifungal metabolites are still understudied (Shahid et al., 2021). Further research should thus be directed at *Bacillus* metabolites as a basis for bio-formulations aiming at combining fertilization, disease control and defense induction, for an integrated management of crops of commercial interest.

Beneficial interactions between plants and their rhizosphere microbiota involve a complex hormonal signaling crosstalk network that endogenously orchestrate plant development and defense. The most abundant auxin in nature, IAA, regulates root gravitropism, cell division and elongation, and the initiation and emergence of lateral roots. Homeostasis of auxin activity maintains a balance between plant defense response and growth, and is modulated through its biosynthesis, conjugation, oxidation, and transport (Rosquete et al., 2012). In this context, the use of *Arabidopsis* mutants, which are affected in perception, signal transduction and gene activation in response to auxin and some other hormones involved in growth regulation and defense activation, has been widely employed to study the underlying mechanisms of plant responses to PGPR (Fan and Smith, 2021; Santoyo, 2022). Understanding these mechanisms would allow us to select promising bacterial strains that could be used as biofertilizers in other non-host plants (Mayer et al., 2019).

Mexico is the world largest producer and exporter of avocado (FAOSTAT - Food and Agriculture Organization of the United Nations, 2021). However, avocado production is hampered by several phytosanitary limitations that reduce fruit yield and quality, one of the main issues being the incidence of diseases caused by phytopathogens (Guevara-Avendaño et al., 2018). Fast-spreading fungal diseases such as *Fusarium* dieback, mainly caused by *Fusarium euwallaceae* and *F. kuroshium* and vectored by ambrosia beetles *Euwallacea* sp. nr. *fornicatus* and *Euwallacea kuroshio*, have been hindering avocado production in several countries (Eskalen et al., 2013; Freeman et al., 2013; Paap et al., 2018); the detection of *E. kuroshio* in Tijuana, Mexico, has alerted national plant health authorities (García-Ávila et al., 2016). Another widely occurring avocado pathogen is the oomycete *P. cinnamomi*, which causes root rot and constitutes the main limiting factor of orchard production in countries such as Mexico, Australia, South Africa and the United States (Fernández-Pavía et al., 2013; Pagliaccia et al., 2013; Solís-García et al., 2021). Although the application of agrochemicals remains the most frequent approach for the control of both diseases, the development of environmentally friendly alternatives such as the use of microorganisms and their specialized metabolites as biological control agents has recently gained interest (Restrepo et al., 2021; Reverchon et al., 2021). Prospecting for microorganisms that could act as biological control agents is thus necessary, especially considering the existing phytosanitary restrictions for exporting and consuming avocado (Stout et al., 2004).

Our previous studies identified two avocado rhizobacterial strains, *Bacillus* sp. A8a and *Bacillus* sp. HA, with antifungal or anti-oomycete activity *in vitro* (Méndez-Bravo et al., 2018; Guevara-Avendaño et al., 2019). To determine the underlying mechanisms behind these properties, we aimed at (1) characterizing the VOCs and diffusible compounds produced by these two bacterial strains, (2) evaluating the activity of these compounds against *F. solani*, *F. kuroshium*, and *P. cinnamomi*, and (3) determining their potential growth promoting effects and resistance induction in *A. thaliana*.

## 2. Materials and methods

### 2.1. Bacterial and fungal strains

The two bacterial strains used in this study were previously isolated from the rhizosphere of avocado trees (*Persea americana* Mill.). *Bacillus* sp. A8a was isolated from an orchard located in Huatusco, State of Veracruz, Mexico and identified through partial 16S rDNA sequencing as closely related to *Bacillus acidiceler* (Méndez-Bravo et al., 2018). *Bacillus* sp. HA was obtained from an avocado orchard in Escondido, California, United States, and identified as a member of the *Bacillus subtilis* species complex, being phylogenetically close to *Bacillus amyloliquefaciens/Bacillus velezensis* (Guevara-Avendaño et al., 2019). Preliminary screening showed that *Bacillus* sp. A8a was able to promote the growth of *A. thaliana in vitro* and to inhibit *P. cinnamomi* through VOC emission (Méndez-Bravo et al., 2018, 2023). In turn, VOCs emitted by *Bacillus* sp. HA reduced the mycelial growth of *F. solani*, *F. kuroshium* and mycelial density of *P. cinnamomi* in sealed double-plate assays (Guevara-Avendaño et al., 2019).

Due to restricted access to the phytopathogenic fungus *F. kuroshium*, and since *F. kuroshium* belong to the *F. solani* species complex (Na et al., 2018), a strain of *F. solani* was used as a proxy for our screenings. The *F. solani* strain LAT-059 was provided by Dr. Mauricio Luna-Rodríguez (Universidad Veracruzana, Mexico) and maintained on potato dextrose agar (PDA, Sigma-Aldrich) at 30°C. The antifungal activity of the selected bacterial strains and compounds was then corroborated against *F. kuroshium* HFEW-16-IV-019, at the quarantine facilities of CNRF [*Centro Nacional de Referencia Fitosanitaria*, SENASICA (*Servicio Nacional de Sanidad, Inocuidad y Calidad Agroalimentaria*), Tecámac, Mexico]. The strain of *P. cinnamomi* was provided by Dr. Alfonso Méndez-Bravo (ENES Morelia, UNAM) and maintained on V8 juice agar (V8 El Original®, Campbell) at 27°C.

### 2.2. *In vitro* evaluation of the antagonistic activity of bacterial VOCs and diffusible compounds against *Fusarium* spp. and *Phytophthora cinnamomi*

Bacterial strains A8a and HA were streaked onto Luria Bertani (LB) agar plates and incubated at 30°C during 24–48 h, while the phytopathogens were grown on PDA agar at their previously mentioned optimum growth temperature for 7 days prior to implementing the antagonism assays. The antagonistic activity of bacterial VOCs was assessed by using the two-sealed-plates method as described previously (Guevara-Avendaño et al., 2019). Briefly, each bacterial strain was streaked in a Petri plate containing LB agar; afterwards, the plate lid was removed and replaced by a Petri plate containing PDA inoculated with a mycelial disc (5 mm of diameter) of *F. solani*, *F. kuroshium*, or *P. cinnamomi* in its center. Then, Petri plates were sealed with plastic wrap (Kleen-pack®) and incubated for 7 days at 30, 27, or 23°C, to evaluate the effect of temperature on VOC emission. Three replicates were performed per assay and as a control, assays were established with LB agar without bacterial strain. The percentage of inhibition (PI) was measured using the formula:

PI = $\left[\frac{D-d}{D}\right]\times 100$

where D is the diameter of mycelial growth in the control and d is the diameter of mycelial growth exposed to bacterial VOCs.

The activity of bacterial diffusible compounds was evaluated in dual culture antagonism assays, as described by Guevara-Avendaño et al. (2018). One disc of 5 mm of diameter was taken from the border of the fungus mycelial growth and placed in the center of a Petri plate containing PDA, then each bacterial strain was taken from a single pure colony with a sterile toothpick and inoculated at a 2 cm distance from the mycelial disc. A sterilized toothpick mark was used as a control. Assays were carried out in triplicate and incubated for 7 days at different temperatures, according to the optimal temperature growth of the pathogen: *F. solani* at 30°C, and *F. kuroshium* and *P. cinnamomi* at 27°C. The antagonistic activity of *Bacillus* sp. A8a against *P. cinnamomi* was previously evaluated by Méndez-Bravo et al. (2018). The PI was calculated using the following formula:

PI = $\left[\frac{R-r}{R}\right]\times 100$

where R is the radial growth of the fungus toward the control treatment, and r is the radial growth of the fungus toward the bacterial treatment.

### 2.3. Analysis of bacterial VOCs by SPME–GC–MS

The VOCs emitted by bacterial strains A8a and HA were characterized by solid phase microextraction (SPME) coupled to gas chromatography and mass spectrometry (GC–MS), following the technique described in our previous study (Guevara-Avendaño et al., 2019). Some modifications were implemented to update previously obtained compound lists (Méndez-Bravo et al., 2018; Guevara-Avendaño et al., 2019) and to assess VOC profiles at different temperatures. Each bacterial strain was re-streaked onto LB agar plates in triplicate, sealed with plastic wrap (Kleen-pack®) and incubated at 30, 27, and 23°C for 7 days. As a control, LB agar plates without bacteria were used. Subsequently, bacterial VOCs were collected with SPME fibers (50–30 μm DVB/CAR/PDMS, Supelco, Inc., Bellefonte, PA), which were inserted into the headspace of the LB agar plates for 1 h. After this time, SPME fibers were injected into the GC port of a gas chromatograph (Perkin Elmer, Clarus 680) coupled to a mass spectrometer (Perking Elmer, Clarus Single Quadrupole [SQ] 8T MS) and VOCs were desorbed at 250°C for 20 min. A splitless injector was used to introduce the sample to the GC column. Helium gas was used as carrier gas (1.0 ml min^−1^, constant flow) and an Elite 5MS column (30 m length × 0.25 mm inner diameter × 0.25 μm film thickness; Perkin Elmer Inc.) was used as a stationary phase. The operational conditions were the following: initial oven temperature of 40°C for 3 min, increased to 160°C (15°C per min), and further increased to 250°C (10°C per min) for a total run time of 20 min. The mass spectrometer was operated in the electron ionization mode at 70 eV with a source temperature of 230°C, and with a continuous scan from 35 to 500 m/z. The mass spectra, retention times, reverse and forward fit values (similarity values) were compared with those reported in the NIST/EPA/NIH Mass Spectrometry Library 2014 (National Institute of Standards and Technology, www.nist.gov) with the Turbomass v. 6.0.0 software (Perkin-Elmer Inc.). The relative abundances of the putatively annotated VOCs were expressed as adjusted peak area and corrected by taking into consideration the area of the compounds that were detected in controls. The identity of some tentatively identified VOCs was subsequently corroborated through co-analysis with volatile compounds purchased in the form of commercial standards (Sigma-Aldrich).

### 2.4. *In vitro* evaluation of the antagonistic activity of selected synthetic VOCs

Eight VOCs produced by bacterial strains A8a and HA and identified through GC–MS analysis were selected to be evaluated in the form of commercial standards against *Fusarium* spp. and *P. cinnamomi*. The selected compounds were: 1-butanol, isobutyl alcohol, 3-methyl-1-butanol, 2,3,5-trimethylpyrazine, DMDS, 2-hexanone, 2-nonanone and acetoin (3-hydroxybutanone), all purchased from Sigma-Aldrich (St. Louis, MO, United States). Compounds were dissolved in different vehicles such as H_2_O (for acetoin, isobutyl alcohol, 1-butanol, 2,3,5-trimethylpyrazine, 3-methyl-1-butanol), H_2_O-Methanol (50:50, v/v; for 2-nonanone, 2-hexanone), and H_2_O-Ethanol (50:50, v/v; for DMDS). The antagonistic assays were carried out by using the two-sealed-plates method as described previously for the assessment of antifungal activity exhibited by bacterial VOCs (Guevara-Avendaño et al., 2019), with some modifications: instead of the bacterial culture, a sterile filter paper (2 cm^2^) with 100 μl of compound was placed in the center of the Petri plate containing LB agar. The concentrations of compounds introduced in the Petri plates corresponded to 0.1, 1, 2, and 3 M. Subsequently, Petri plates were sealed with plastic wrap (Kleen-pack®) and incubated for 7 days at 30°C (*F. solani*) and 27°C (*F. kuroshium* and *P. cinnamomi*). Each assay was replicated three times and control assays were established with LB agar and with the vehicle where the pure standard was dissolved. Afterwards, the PI was calculated with the formula previously described for bacterial VOCs (section 2.2. of this article).

### 2.5. Obtention of bacterial crude extracts

The analysis of bacterial diffusible compounds was carried out by obtaining crude extracts of bacterial strains A8a and HA, following the method described in our previous studies (Báez-Vallejo et al., 2020; Guevara-Avendaño et al., 2020). Briefly, bacterial strains were re-streaked onto LB agar plates and incubated at 30°C during 24–48 h. Subsequently, a bacterial suspension was prepared in sterile distilled water at a concentration of 1.5 × 10^8^ CFU (Colony Forming Units) ml^−1^, using the McFarland scale, in a UV–Vis spectrophotometer (550 nm, Eppendorf BioSpectrometer® Basic). Two milliliters of bacterial dilution were then added for each liter of LB broth and incubated at 30°C for 7 days under constant agitation at 180 rpm; the same procedure was carried out for the control, which was prepared from LB broth and 2 ml of sterile distilled water. After incubation, bacterial cultures were centrifuged at 20,000 × *g* for 15 min at 25°C to separate the supernatant from the cell biomass. Starting from the supernatant, the crude extracts were obtained by liquid–liquid extractions (1:1, v/v) and each extraction was conducted five times with each solvent, first with ethyl acetate (EtOAc) to extract the non-polar and medium polar compounds, and subsequently with n-butanol (n-BuOH) for extraction of the compounds of greater polarity. Organic phases were filtered through a layer of anhydrous sodium sulfate and the solvent excess was removed by evaporation under reduced pressure in a Rotavapor® (BUCHI, RII, Switzerland), until obtaining the crude extracts of EtOAc and n-BuOH. Finally, 50 mg of each crude extract were dissolved in 1 ml of methanol with formic acid (0.1%, v/v) to be used for the tentative identification of diffusible compounds, using ultra-high performance liquid chromatography (UPLC) and high-resolution mass spectrometry (HRMS).

### 2.6. *In vitro* evaluation of the antagonistic activity of bacterial crude extracts

The inhibitory effect of the bacterial crude extracts from strains A8a and HA on the mycelial growth of *F. solani*, *F. kuroshium*, and *P. cinnamomi* was evaluated using the agar well diffusion method as reported by Rizi et al. (2015) and Balouiri et al. (2016), with some modifications. First, different treatments were prepared: (a) bacterial and control crude extracts were dissolved in vehicle (H_2_O-Methanol [7:3]); (b) positive control Thiabendazole (Sigma-Aldrich, St. Louis, MO, United States) was dissolved in H_2_O; (c) negative control was vehicle and H_2_O only; (d) blank control was fungus only. Subsequently, these treatments were added to PDA immediately before being poured into a 12-well plate (Eppendorf® Cell Culture Plates). The treatments (a) and (b) were tested at concentrations ranging from 0.002 to 2 mg ml^−1^ (3 ml per well). Then, an inoculum suspension was prepared from *Fusarium* spp. or *P. cinnamomi* by placing a mycelial plug, previously incubated at their abovementioned optimum growth temperature for 7 days, in a 50 ml centrifuge tube with 10 ml of sterile distilled water. The tube was slightly shaken and the inoculum suspension was poured through a filter paper or sterile gauze grade 1 (Whatman®). For *Fusarium* spp., the number of spores was quantified using a Neubauer chamber (Bright-Line™ Hemacytometer, Sigma-Aldrich), until reaching a concentration of 1.0 × 10^6^ spores ml^−1^. Once PDA solidified, 3 μl of spore suspension were added to the center of each well. For *P. cinnamomi*, 3 μl of mycelium suspension adjusted at 1.0 × 10^6^ mycelium fragments ml^−1^ were used. Six replicates of each concentration were tested. Plates were incubated at 27°C for 72 h, after which the PI of mycelial growth was calculated as indicated above for antagonism assays by bacterial VOCs.

### 2.7. Chemical profiling of antagonistic compounds using UPLC-HRMS

The tentative identification of diffusible compounds in bacterial crude extracts was carried out by UPLC coupled to quadrupole time of flight (QTOF)–HRMS, following the technique described by Guevara-Avendaño et al. (2020) with some modifications. The MS analysis was carried out in a mass range of 100 to 1,600 Da. Data were acquired and processed with MassLynx and MarkerLynx software (version 4.1, Waters). The analysis of the similarity of chemical profiles was carried out by principal component analysis (PCA) using MetaboAnalyst 4.0. Tentative identification of compounds in bacterial extracts was carried out by comparing the obtained and reported mass spectra in databases such as METLIN,^1^ ECMDB,^2^ and ChemSpider,^3^ as well as bibliography.

### 2.8. Microscopic analysis

The hyphal morphology of *F. solani*, *F. kuroshium*, and *P. cinnamomi* exposed to commercial VOCs and crude extracts produced by both bacterial strains was analyzed by Confocal Scanning Laser Microscopy (CSLM) and Scanning Electron Microscopy (SEM) to observe possible structural alterations in the mycelium. Sample preparation was implemented as described by Guevara-Avendaño et al. (2020). Samples for CSLM were fixed in 4% paraformaldehyde +0.2% cacodylate in Phosphate-Buffered Saline (PBS). Samples were stained with 50 μl of calcofluor white (Sigma-Aldrich, 1 g l^−1^) for 15 min. Images were acquired with a Leica TCS-SP8 + STED microscope (Leica Microsystems, Germany) using plan apochromatic 40× (NA 1.25, oil) and 63× (NA 1.4, oil) objectives. Calcofluor-stained samples were recorded in grayscale channel (410–440 nm wavelength emission; excitation wavelength 405 nm).

Samples for SEM were fixed in Karnovsky solution (Electron Microscopy Sciences, United States), rinsed twice in PBS buffer for 5 min, dehydrated in a graded ethanol series (30–100%) during 50 min for each concentration, dried in a Quorum K850 (United Kingdom) critical point drying with CO_2_ and attached to aluminum stubs using a carbon adhesive prior to coating with gold–palladium in a sputtering Quorum Q150 (United Kingdom). The preparations were observed and photographed with a FEI™ Quanta 250-FEG microscope (Czech Republic) with a 5,000× magnification.

Mycelium samples not exposed to bacterial compounds were considered as controls. Three samples per treatment were evaluated, and three to five fields per sample were observed.

### 2.9. Plant material and growth conditions

The plant growth promoting effect of bacterial VOCs and diffusible compounds emitted by strains A8a and HA was evaluated on wild-type *A. thaliana* ecotype Columbia (Col-0) seedlings and on the transgenic line *DR5:GUS* (Ulmasov et al., 1997), indicator of endogenous accumulation of auxins. The phytohormone-related mutants *aux1-7* (Picket et al., 1990), *axr1-3* (Lincoln et al., 1990), *coi1-1* (Xie et al., 1998), *jar1* (Staswick et al., 1992), and *ein2-1* (Guzmán and Ecker, 1990) were evaluated for their responsiveness to bacterial co-culture. Additionally, the transgenic lines *LOX2:GUS* (Schommer et al., 2008), reporter of biosynthesis and activation of response genes to jasmonic acid (JA); and *PR1:GUS* (Beilmann et al., 1992), indicator of the activation of genes by salicylic acid (SA), were used as gene markers to assess the activation of defense pathways ISR (Induced Systemic Resistance) and SAR (Systemic Acquired Resistance), respectively, at the molecular level.

Seeds were sterilized with 95% ethanol for 5 min and 20% sodium hypochlorite for 7 min. After five consecutive washes in distilled water, seeds were vernalized at 4°C for 3 days in darkness and subsequently germinated and grown on agar plates containing 0.2× MS agar (Murashige and Skoog basal salts mixture, PhytoTech Labs.) enriched with 0.6% saccharose and 1% phyto-agar, at pH 5.8. Plates were placed vertically at an angle of 65 degrees in a plant-growth chamber with a photoperiod of 16 h of light, 8 h of darkness, light intensity of 200 μmol m^−2^ s^−1^, at 23°C and 60% relative humidity.

### 2.10. Histochemical analysis

The analysis of seedlings from each *Arabidopsis* transgenic line, exposed to bacterial VOCs and diffusible compounds, was implemented as follows: when concluded the time of exposure, the enzymatic reaction of β-glucuronidase (GUS) activity was carried out by submerging at least five seedlings per treatment in a GUS reaction buffer (X-Gluc, 5-bromo-4-chloro-3-indolyl-β-D-glucuronide; Jefferson, 1987) and incubating overnight at 37°C. The stained seedlings were cleared using the method established by Malamy and Benfey (1997). Finally, a representative seedling was chosen and photographed, using Nomarsky optics on an inverted microscope Leica DMI 6000.

The evaluation of *PR1:GUS* seedlings was implemented in a 12-well plate (Eppendorf® Cell Culture Plates) where 2 ml of 0.2× MS broth were poured in each well. Twenty microliters of each of the following treatments were added: (1) bacterial suspension (1.5 × 10^8^ CFU ml^−1^) of strain A8a; (2) bacterial suspension of strain HA; (3) bacterial suspension of strain A8a + HA, to evaluate a possible synergistic effect of both strains on plant growth promotion. Twenty microliters of 0.2× MS broth and LB broth were used as controls. Subsequently, five seedlings of 15 days after germination (dag) were carefully transplanted in each well, and two replicates were performed per treatment. Finally, 12-well plates were sealed and incubated for 24 h in the plant-growth chamber described previously.

### 2.11. *In vitro* evaluation of growth-promoting effects of bacterial VOCs and diffusible compounds in early development stages of *Arabidopsis thaliana*

The growth promoting effect of bacterial VOCs produced by strains A8a and HA were evaluated on early development stages of *A. thaliana* using divided Petri dishes. A bacterial suspension (1.5 × 10^8^ CFU ml^−1^) of each strain was prepared in LB broth; the suspension was prepared from a 24-h bacterial culture and the concentration was estimated based on the McFarland scale. Twenty microliters of bacterial suspension were placed in one of the compartments of the Petri dish containing LB agar, while eight seedlings of seven dag were transplanted in the other compartment containing 0.2× MS agar, as described by Santoro et al. (2016). The plant growth promoting activity of bacterial diffusible compounds from both strains was evaluated *in vitro* as described by Méndez-Bravo et al. (2018), with some modifications. Briefly, eight seedlings (7 dag) were transplanted into a Petri dish containing 0.2× MS agar, then 500 μl of a bacterial suspension (1.5 × 10^8^ CFU ml^−1^) were inoculated at 2.5 cm from the root tips. Seedlings without bacterial strains were considered as a control. Four replicates were set up per treatment. Petri dishes were sealed and incubated in a plant-growth chamber as described in the previous section for 7 days.

At the end of the incubation period, root system architecture and biomass were analyzed was follows: primary root length of at least 24 seedlings per treatment was measured with a Vernier caliper; lateral roots were counted under a stereo microscope; for root hairs, digital images were taken using a stereo microscope and a portion of 2 cm from the primary root tip was used for analysis by ImageJ (v. 1.8.0_112) to determine the number and length of root hairs; fresh weight was measured on an analytical scale.

### 2.12. Long-term growth promoting activity of bacterial strains in *Arabidopsis thaliana*, with and without *Fusarium solani*: a pot experiment

The *A. thaliana* ecotype Columbia (Col-0) was used to evaluate the long-term plant growth promoting activity of bacterial strains A8a and HA, inoculated individually or in combination, as well as their *in vivo* antagonistic activity against *F. solani*. The *A. thaliana* seeds used in these assays were subjected to the sterilization and vernalization treatment detailed above, and were germinated onto MS Petri plates, incubated for 7 days in a plant-growth chamber under the same conditions as previously described.

First, we evaluated the plant growth promotion effect of the tested bacterial inoculants in a pot experiment, as follows: MS plates with 10 *A. thaliana* seedlings (7 dag) were inoculated with one the following treatment: (1) strain A8a; (2) strain HA; (3) A8a + HA. Controls consisted in plants without bacterial inoculant. Two MS plates were established per treatment. Inoculation was carried out by preparing suspensions with bacterial biomass from 24 -h-old cultures grown in LB at 25°C, resuspended in sterilized water to reach a concentration of 1.5 × 10^8^ CFU ml^−1^, according to the 0.5 standard of the McFarland scale. Treatment A8a + HA was prepared by mixing 1:1 (v:v) bacterial suspensions of A8a and HA strains. Five hundred microliters of suspension were streaked onto the MS medium at 2.5 cm from the root tips. Subsequently, plants were allowed to grow for 14 more days under the same incubation conditions. Then, 10 plants per treatment were carefully transplanted to plastic pots (pot volume of 163 cm^3^, one plant per pot) containing an autoclaved (120°C for 1 h) composite substrate, which was prepared by mixing peat moss, vermiculite and perlite (proportion: 3, 1, 1, respectively). Substrate was moistened with sterilized water at field capacity, and supplemented with 23 ml of a fertilizer solution at 0.5 g l^−1^ (fertilizer: Water Soluble All Purpose, Plant Food Miracle Gro®). Plants were allowed to acclimate for 1 day. Next, plants were inoculated again with bacterial suspensions prepared as described previously. Each plant was inoculated around the base of the stem with 6 ml of bacterial suspension from their respective treatment; for control plants, 6 ml of sterilized water were applied. The number of leaves and fresh weight of aerial part of plants were measured at 29 days after inoculation. Subsequently, aerial parts were dried in an oven (H-62, BG) at 65°C for 72 h, after which dry weight was recorded. Plants were kept in growth chamber during all experiment.

In a second assay, we evaluated the *in vivo* biocontrol effect of bacterial strains and their combination in *A. thaliana* infected with *F. solani*. Sixty plants (12 dag) were individually transplanted to pots (163 cm^3^) containing the same sterilized composite substrate as described above. Plants were acclimatized for 1 day and subsequently inoculated with 6 ml of one of the following treatments: (1) bacterial suspension (1.5 × 10^8^ CFU ml^−1^) prepared from strain A8a, as described above; (2) bacterial suspension from strain HA, (3) bacterial suspension from A8a + HA, and (4) sterilized water (control). Inoculations were carried out at two, seven and 14 days after pot transplantation. A total of 15 plants were used per treatment. Seven days after the last inoculation of bacterial suspension, four leaves per plant were pricked at the apical area with a syringe, and inoculated with 10 μl of *F. solani* spore suspension (concentration: 1 × 10^6^ spores ml^−1^). Control plants were inoculated with water. Sixteen days after the infection, the percentage of damaged leaf area was recorded.

### 2.13. Statistical analysis

Data from the *in vitro* antagonism assays and *in vitro* plant growth promotion assays were analyzed using the RStudio® software (version 1.1.463, Windows NT 6.1, WOW64). Results from the *in vivo* experiment were analyzed in Minitab® software (version 19.1.1). Data normality was checked with the Kolmogorov–Smirnov test. A one-way analysis of variance (ANOVA) followed by a Tukey’s post-hoc test was implemented for normally distributed data. Data that did not meet a normal distribution was analyzed with the non-parametric Kruskal-Wallis test. All statistical tests were considered significant at *p* ≤ 0.05.

## 3. Results

### 3.1. Antagonistic activity of bacterial VOCs and compound identification

Temperature was an important factor in the emission of antimicrobial VOCs, since no growth inhibition was detected at 23°C, for neither of the three tested pathogens. Similarly, no significant inhibition of *F. solani* by bacterial VOCs was registered, regardless of the assay temperature (Supplementary material 1). In contrast, the VOCs emitted by *Bacillus* sp. A8a and *Bacillus* sp. HA significantly inhibited the growth of *F. kuroshium* at 27°C whilst only VOCs from strain A8a were able to significantly reduce the growth of *P. cinnamomi* at that temperature (Supplementary material 1).

Although VOCs emitted by the two bacterial strains were reported in our previous studies (VOCs from *Bacillus* sp. A8a at 27°C, Méndez-Bravo et al., 2018; VOCs from *Bacillus* sp. HA at 30°C, Guevara-Avendaño et al., 2019), we describe here the chemical analysis of VOCs emitted at the three tested incubation temperatures and used commercial standards when available to corroborate the tentative compound identification. In total, 32 compounds were tentatively identified, belonging principally to the ketone, alcohol, pyrazine, ester and sulfur-compound functional groups (Table 1). For each bacterial strain, the VOC profiles were more diverse at 27°C and 30°C than at 23°C, which corresponds to the temperatures at which higher inhibitions of pathogen growth were observed. Some of the unequivocally identified compounds include 1-butanol and 3-methyl-1-butanol, which were emitted by both strains. Compounds which were only detected at 27°C (temperature at which significant inhibition of *F. kuroshium* or *P. cinnamomi* was registered) include 2-methyl-1-butanol (strain A8a), and 2-octanone (strain HA; Table 1).

**Table 1:** Chemical composition of VOCs emitted by *Bacillus* sp. A8a and *Bacillus* sp. HA at different temperatures, tentatively identified through SPME-GC–MS.

Compound identity	RT (min)	RA (%)			Functional group	Bacterial strain
		30°C	27°C	23°C		
**Isobutyl alcohol**	2.40	2.31 ± 1.30^a^ (68.77 ± 11.76)	1.11 ± 0.69^a^ (65.35 ± 16.90)	2.65 ± 1.54^a^ (80.95 ± 7.71)	Alcohol	A8a
**1-Butanol**	2.79	9.52 ± 1.82^a^ (27.03 ± 5.98)	8.23 ± 1.76^a^ (23.50 ± 7.62)	10.07 ± 1.35^a^ (43.23 ± 18.60)	Alcohol	A8a
		13.16 ± 1.00^b^ (55.33 ± 15.86)	13.85 ± 1.52^b^ (62.13 ± 2.19)	56.67 ± 5.29^a^ (60.57 ± 10.84)		HA
Methyl thioacetate	3.28	0^b^	0^b^	4.13 ± 2.07^a^ (51.60 ± 11.27)	Thiol	A8a
**Acetoin**	3.41	0.94 ± 0.19^b^ (82.30 ± 11.52)	0.60 ± 0.36^c^ (85.20 ± 1.27)	2.65 ± 1.35^a^ (80.40 ± 11.04)	Ketone	A8a
**3-Methyl-1-butanol**	3.87	0^c^	3.33 ± 0.63^a^ (48.97 ± 3.67)	1.46 ± 0.38^b^ (48.80 ± 1.48)	Alcohol	A8a
		0^b^	0^b^	5.59 ± 1.92^a^ (29.17 ± 23.02)		HA
Methyl isobuthyl ketone	3.93	17.76 ± 1.01^a^ (71.80 ± 2.72)	19.93 ± 2.72^a^ (72.60 ± 2.52)	11.02 ± 3.43^b^ (48.60 31.83)	Ketone	HA
**2-Methyl-1-butanol**	3.94	0^b^	1.78 ± 0.58^a^ (19.52 ± 17.49)	0^b^	Alcohol	A8a
**Dimethyl disulfide**	4.01	14.74 ± 1.20^a^ (96.10 ± 0.69)	12.94 ± 4.12^ab^ (97.13 ± 0.15)	8.68 ± 1.89^b^ (97.43 ± 0.23)	Sulfur	A8a
**3-Methyl-2-pentanone**	4.14	5.62 ± 1.08^a^ (80.97 ± 7.12)	6.65 ± 2.39^a^ (81.13 ± 8.71)	6.84 ± 1.78^a^ (82.40 ± 4.78)	Ketone	A8a
2,3,4,5-Tetrahydropyridine	4.16	45.71 ± 1.38^a^ (12.80 ± 0.99)	0^c^	8.13 ± 5.11^b^ (76.95 ± 3.32)	Amine	HA
**2-Hexanone**	4.75	3.68 ± 0.61^b^ (66.70 ± 5.57)	5.30 ± 0.19^a^ (71.83 ± 4.56)	2.98 ± 0.94^b^ (56.43 ± 3.61)	Ketone	HA
2-Methyl-3-pentanol	4.93	0^b^	0^b^	0.83 ± 0.29^a^ (10.41 ± 4.86)	Alcohol	A8a
2-Hydroxy-3-pentanone	5.05	0^b^	0^b^	0.75 ± 0.25^a^ (63.80 ± 4.06)	Ketone	A8a
2-Propenoic acid	5.68	0^b^	0.63 ± 0.42^a^ (62.85 ± 0.07)	0^b^	Ester	A8a
3-Methylbutanal oxime	5.81	1.67 ± 0.16^a^ (57.53 ± 8.60)	1.19 ± 0.14^b^ (62.63 ± 7.58)	0^c^	Oxime	HA
5-Methyl-2-hexanone	5.86	0^b^	0^b^	2.08 ± 0.25^a^ (71.87 ± 3.35)	Ketone	A8a
Methyl isoamyl ketone	5.86	0^b^	0.75 ± 0.52^a^ (64.80 ± 5.37)	0^b^	Ketone	A8a
		2.23 ± 0.46^b^ (58.73 ± 3.79)	3.58 ± 0.28^a^ (61.73 ± 2.89)	3.93 ± 2.37^a^ (55.75 ± 9.69)		HA
3,3-Dimethylbutan-2-yl-2-methylbutanoate	7.12	2.23 ± 0.32^a^ (10.83 ± 1.33)	2.17 ± 0.13^a^ (11.48 ± 6.21)	0^b^	Ester	A8a
2-Methyl-6-heptanone	7.27	0^b^	0^b^	0.82 ± 0.15^a^ (72.43 ± 13.77)	Ketone	A8a
		2.71 ± 0.25^a^ (85.47 ± 2.40)	3.35 ± 0.51^a^ (83.50 ± 5.86)	0^b^		HA
5-Methyl-2-heptanone	7.40	4.68 ± 0.37^b^ (77.67 ± 4.44)	6.74 ± 0.80^a^ (73.73 ± 7.09)	0^c^	Ketone	HA
**2-Octanone**	7.74	0^b^	3.14 ± 0.39^a^ (75.20 ± 7.47)	0^b^	Ketone	HA
**2, 3, 5-Trimethylpyrazine**	7.90	6.86 ± 1.16^a^ (81.13 ± 5.05)	6.95 ± 0.91^a^ (78.80 ± 11.91)	9.19 ± 2.48^a^ (87.50 ± 2.50)	Pirazine	A8a
Benzyl alcohol	8.31	0^b^	0.94 ± 0.26^a^ (47.63 ± 5.12)	0^b^	Alcohol	A8a
**2-Nonanone**	8.53	1.06 ± 0.06^a^ (49.87 ± 4.85)	1.33 ± 0.23^a^ (53.60 ± 4.43)	1.39 ± 0.85^a^ (49.73 ± 6.93)	Ketone	HA
**2-Ethyl-3,6-dimethylpyrazine**	8.80	0.62 ± 0.36^b^ (56.60 ± 16.83)	0.37 ± 0.04^c^ (67.43 ± 4.02)	1.00 ± 0.41^a^ (47.93 ± 15.77)	Pirazine	A8a
**2-Ethyl-3,5-dimethylpyrazine**	8.87	0.51 ± 0.30^a^ (33.05 ± 20.29)	0.53 ± 0.08^a^ (25.57 ± 9.17)	0^b^	Pirazine	A8a
2-Methyl-6-[(1Z)-1-propenyl]pyrazine	9.05	0.50 ± 0.03^a^ (25.03 ± 2.48)	0.45 ± 0.05^a^ (22.80 ± 4.15)	0^b^	Pirazine	A8a
3,5-Dimethyl-2-octanone	9.08	0.86 ± 0.03^a^ (42.43 ± 14.34)	0.92 ± 0.14^a^ (31.27 ± 8.03)	0^b^	Ketone	HA
Phenylethyl alcohol	9.24	1.52 ± 0.48^a^ (53.77 ± 17.83)	0.85 ± 0.29^b^ (70.40 ± 13.19)	0^c^	Alcohol	A8a
2-Ethylhexanal	9.51	0.36 ± 0.07^a^ (15.17 ± 3.91)	0.29 ± 0.05^a^ (10.43 ± 9.97)	0^b^	Aldehyde	HA
2-Decanone	9.72	1.08 ± 0.20^b^ (53.03 ± 2.40)	1.39 ± 0.25^b^ (54.10 ± 1.90)	1.70 ± 0.98^a^ (30.25 ± 3.89)	Ketone	HA
Acetophenone, 2′-amino-	11.21	0.40 ± 0.23^a^ (33.60 ± 1.27)	0^b^	0^b^	Ketone	HA

Numbers between brackets indicate similarity percentage ± standard deviation. RT: retention time. RA: relative abundance ± standard deviation. Bold compounds indicate compounds which identity was corroborated through the use of commercial standards. Different letters indicate significant differences between temperatures for the same compound (Kruskal-Wallis, *p* ≤ 0.05).

**Figure 1:**
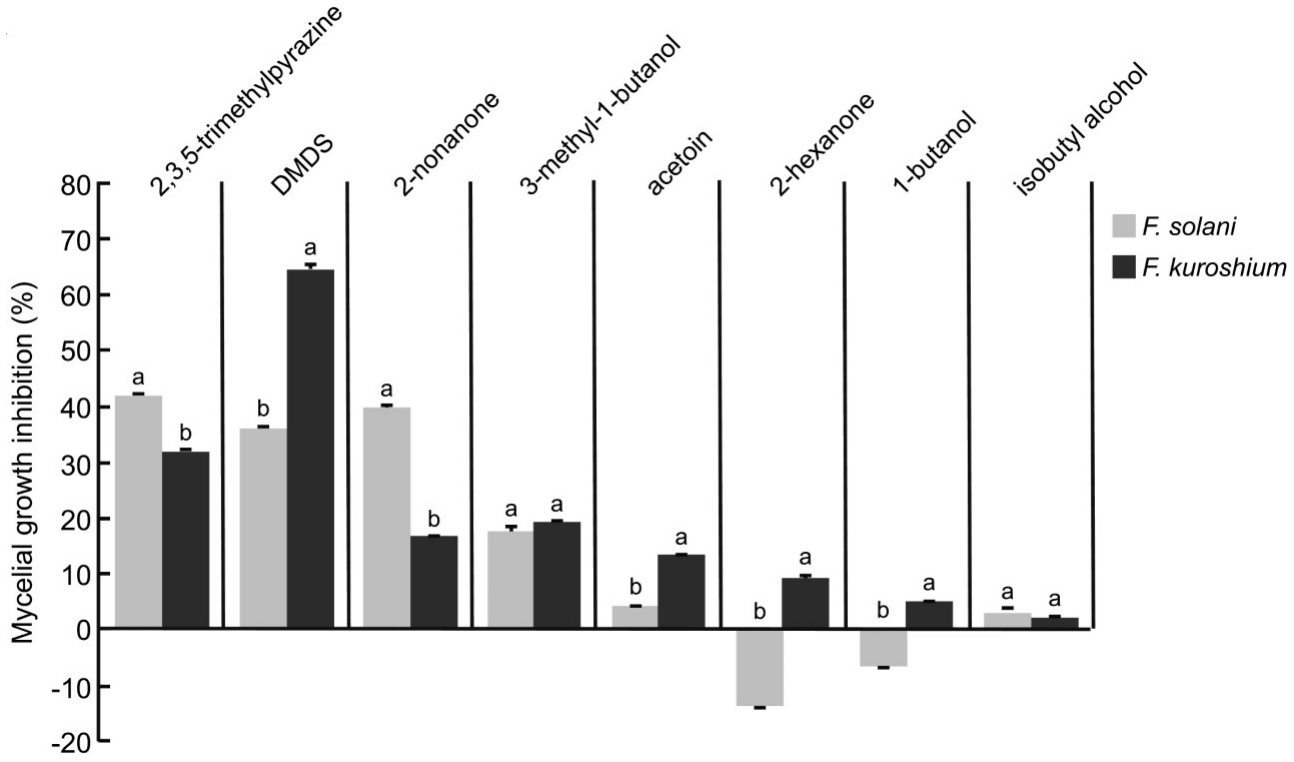
Antifungal activity of commercial VOCs against *F. solani* and *F. kuroshium*, indicated as percentages of inhibition of mycelial growth by commercial VOCs at a 3 M concentration. Values indicate average ± standard deviation (*n* = 3). Letters indicate significant differences between *Fusarium* species for each compound (Student *t* test, *p* ≤ 0.05).

Compounds presenting a large relative abundance and being commercially available were selected to be tested in the form of commercial standards against *F. solani* and *F. kuroshium* (Figure 1). Standards could not be evaluated against *P. cinnamomi* due to the limited compound quantity that was available. The 3 M concentration was determined based on a preliminary concentration-dependent assay (data not shown). Although the magnitude of the observed inhibition depended on the *Fusarium* species under study, four compounds caused the main reduction in mycelial growth in both pathogens: 2,3,5-trimethylpyrazine (emitted by strain A8a), DMDS (strain A8a), 2-nonanone (strain HA) and 3-methyl-1-butanol (emitted by both strains). The largest inhibition of *F. solani* was induced by 2,3,5-trimethylpyrazine (42.2%) while DMDS reduced *F. kuroshium* mycelial growth by 59.3%. Interestingly, 2-hexanone and 1-butanol inhibited the growth of *F. kuroshium* while stimulating that of *F. solani*. The CSLM images evidenced the morphological alterations caused by some of the commercial VOCs on *Fusarium* spp. hyphae, in the form of small conglobations and thinning of hyphal tips (2-hexanone, 2,3,5-trimethylpyrazine and 2-nonanone) or thickening of the middle section of hyphae (DMDS; Supplementary material 2).

### 3.2. Antagonistic activity of bacterial diffusible compounds and compound identification

No significant inhibition of *F. solani* or *F. kuroshium* mycelial growth by *Bacillus* spp. A8a or HA was detected in dual culture assays. The only significant reduction of mycelial growth was observed in *P. cinnamomi* in dual culture with *Bacillus* sp. A8a (19%, Supplementary material 3).

Crude extracts were obtained from both bacterial strains in EtOAc and n-BuOH, extract yields being larger in the latter solvent (≥2.00 g l^−1^, as opposed to approximately 0.20 g l^−1^ for EtOAc extracts). When tested against the three pathogens, the antifungal activity of EtOAc crude extracts was larger than that of n-BuOH crude extracts (2 mg ml^−1^) for both bacterial strains (Table 2). No significant inhibition of mycelial growth was observed at lower concentrations (0.002, 0.02 and 0.2 mg ml^−1^, data not shown). The largest inhibition percentages were obtained for EtOAc crude extracts from *Bacillus* sp. A8a against *P. cinnamomi*, *F. kuroshium*, and *F. solani* (100.0, 77.2 and 32.4%, respectively). Interestingly, a loss of pigmentation of *F. kuroshium* mycelium was observed when in contact with EtOAc and n-BuOH extracts (Supplementary material 4).

**Table 2:** Percentages of inhibition of *F. solani*, *F. kuroshium*, and *P. cinnamomi* mycelial growth by crude extracts from *Bacillus* sp. A8a and *Bacillus* sp. HA at 2  mg  ml^−1^.

			Percentage of inhibition (%)			
	Extract		Treatment	Fungus growth control	Control (−) H_2_O:MeOH	Control (+) Thiabendazole
*F. solani*	EtOAc	Control	0.00 ± 0.02	0.00 ± 0.03	0.00 ± 0.03	100 ± 0.00*
		A8a	32.39 ± 0.01*			
		HA	13.74 ± 0.03*			
	n-BuOH	Control	0.00 ± 0.02	0.00 ± 0.02	0.00 ± 0.03	100 ± 0.00*
		A8a	0.92 ± 0.02*			
		HA	0.00 ± 0.02			
*F. kuroshium*	EtOAc	Control	0.00 ± 0.01	0.00 ± 0.03	0.00 ± 0.03	100 ± 0.00*
		A8a	77.20 ± 0.00*			
		HA	6.16 ± 0.03*			
	n-BuOH	Control	0.00 ± 0.02	0.00 ± 0.01	0.00 ± 0.01	100 ± 0.00*
		A8a	0.00 ± 0.03			
		HA	0.00 ± 0.03			
*P. cinnamomi*	EtOAc	Control	0.00 ± 0.00	3.17 ± 2.63	0.00 ± 0.00	60.71 ± 10.08*
		A8a	100 ± 0.00*			
		HA	4.76 ± 2.74			
	n-BuOH	Control	0.00 ± 0.00	3.17 ± 2.63	0.00 ± 0.00	60.71 ± 10.08*
		A8a	16.66 ± 3.07*			
		HA	6.35 ± 6.68			

Values represent average ± standard deviation (*n* = 6). Fungus growth control corresponds to the fungus growing in PDA. Control (−) corresponds to the vehicle (H_2_O:MeOH). Control (+) corresponds to the commercial fungicide thiabendazole.

* Indicates significant differences with negative control (H_2_O:MeOH; Kruskal-Wallis, *p* ≤ 0.05).

**Figure 2:**
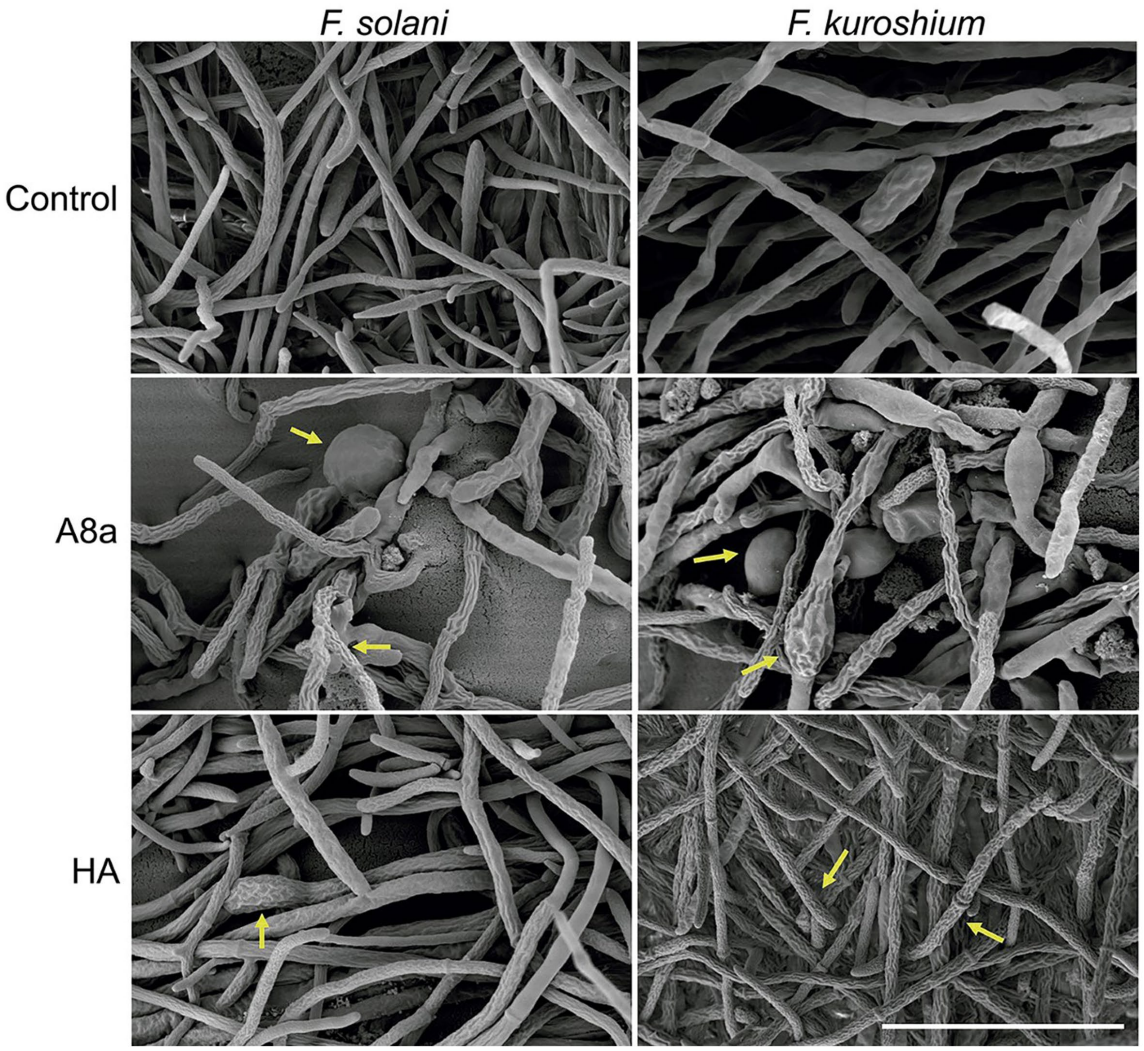
SEM images of *F. solani* and *F. kuroshium* hyphae exposed to EtOAc extracts from *Bacillus* sp. A8a and *Bacillus* sp. HA during 3  days. Scale bar: 40 μm (5000×). Yellow arrows highlight morphological deformations.

The potential alterations induced in hyphae by treatments exhibiting significant mycelial growth inhibition were further documented by SEM. The SEM analysis of *Fusarium* spp. hyphae exposed to EtOAc extracts from both bacterial strains evidenced morphological deformations compared with hyphae in the control treatment (Figure 2). Extracts from *Bacillus* sp. HA induced a coarse texture and loss of turgescence in hyphae, whilst extracts from *Bacillus* sp. A8a caused the appearance of conglobations and hyphal distortions in both fungi. As *P. cinnamomi* did not grow when exposed to EtOAc extracts from *Bacillus* sp. A8a, no SEM analysis could be performed.

EtOAc crude extracts from both bacterial strains, which exhibited the strongest antifungal and anti-oomycete activity, were subsequently subjected to UPLC-QTOF-MS for analysis (Table 3). The tentative identification of compounds detected in EtOAc extracts from *Bacillus* sp. A8a and *Bacillus* sp. HA revealed the presence of polyketides such as macrolactins, difficidin and bacillaene, non-ribosomal peptides such as bacilysin, and the growth regulator IAA.

**Table 3:** Tentative identification of compounds detected in EtOAc extracts from *Bacillus* sp. A8a and *Bacillus* sp. HA by UPLC–QTOF–MS.

Bacterial strain	RT (min)	m/z (observed)	Compound	Molecular formula	Adduct	Calculated adduct m/z	Mass error (ppm)	Fragments	
A8a	0.54	291.0981	Bacilysin	C_12_H_18_N_2_O_5_	[M + Na–2H]^−^	291.0957	8.2	200.0459	153.0662
	0.79	174.0555	Indole acetic acid	C_10_H_9_NO_2_	[M–H]^−^	174.0555	0	144.0446	92.0497
	4.91	441.2086	Macrolactin A	C_24_H_34_O_5_	[M + K]^+^	441.2043	9.7	297.1702	251.1265
	6.03	617.2949	Bacillaene	C_34_H_48_N_2_O_6_	[M + K–2H]^−^	617.2993	−7.1	299.1518	265.1476
	10.86	367.23	Macrolactin N	C_24_H_34_O_4_	[M–H_2_O–H]^−^	367.2273	7.3	339.1960	325.1804
HA	0.62	269.1149	Bacilysin	C_12_H_18_N_2_O_5_	[M–H]^−^	269.1137	4.4	241.1195	197.1296
	0.79	174.0555	Indole acetic acid	C_10_H_9_NO_2_	[M–H]^−^	174.0555	0	144.0449	92.0501
	3.08	545.3032	Difficidin	C_31_H_45_O_6_P	[M + H]^+^	545.3032	0	360.1902	211.0848
	14.66	481.3343	Macrolactin U	C_31_H_44_O_4_	[M + H]^+^	481.3318	5.2	429.3367	395.2982

m/z: mass/charge; RT: retention time (min).

### 3.3. Growth promotion of *Arabidopsis* induced by bacterial diffusible compounds and VOCs

**Figure 3:**
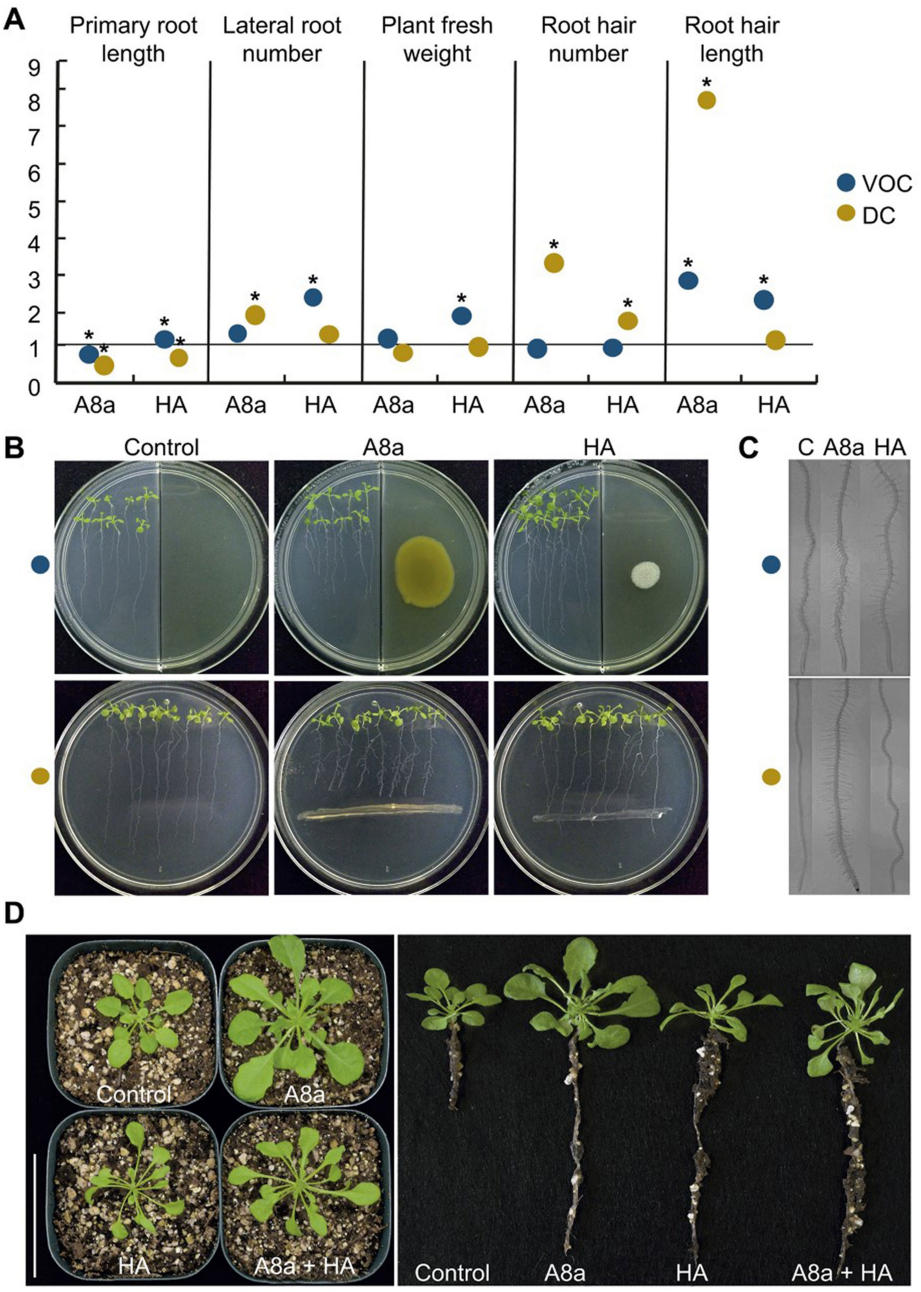
Effects of bacterial inoculation and VOC emission by strains *Bacillus* spp. A8a and HA on growth and development of *Arabidopsis thaliana* (Col-0). **(A)** Measured growth and developmental parameters on 14  day-old seedlings co-cultured with bacterial strains. Measurements were normalized versus control; asterisks mean statistically significant differences (one-way ANOVA, Tukey test, *p* ≤ 0.05, *n* = 24–30). **(B)** Representative images of seedlings co-cultured or exposed to bacterial VOCs for 7  days. **(C)** Photographs of root hairs developed on primary roots. **(D)** Long-term effects of bacterial strains on *Arabidopsis* plants after 29  days co-cultured in soil. Representative photographs show the plant growth promoting effect of bacterial inoculation on shoot and root development (*n* = 10); the assay was replicated three times.

The effect of bacterial inoculation and VOC emission on early *A. thaliana* seedling growth *in vitro* was evaluated on standard or physically divided Petri dishes, respectively. At 7 days after co-cultivation, bacterial diffusible and volatile compounds induced morphological changes in the root architecture by significantly shortening the primary root, increasing development of lateral roots, and stimulating root hair formation and elongation (Figures 3A–C).

Growth promotion of *A. thaliana* by bacterial strains, inoculated individually or in combination in a pot experiment, was also evident at late developmental stages. After 29 days of substrate inoculation, *Bacillus* sp. A8a increased the number of leaves by up to 55%, stimulated root development and induced a five-fold biomass accumulation, reflected by the increase in aerial fresh and dry weight of inoculated plants as compared with control plants (Figure 3D). The inoculation of *Bacillus* sp. HA and combination of both strains caused a moderate effect on growth stimulation, compared with the effects induced by inoculation with single strain A8a (Figure 3D; Supplementary material 5).

### 3.4. Plant growth promotion by *Bacillus* spp. A8a and HA involves auxin signaling

**Figure 4:**
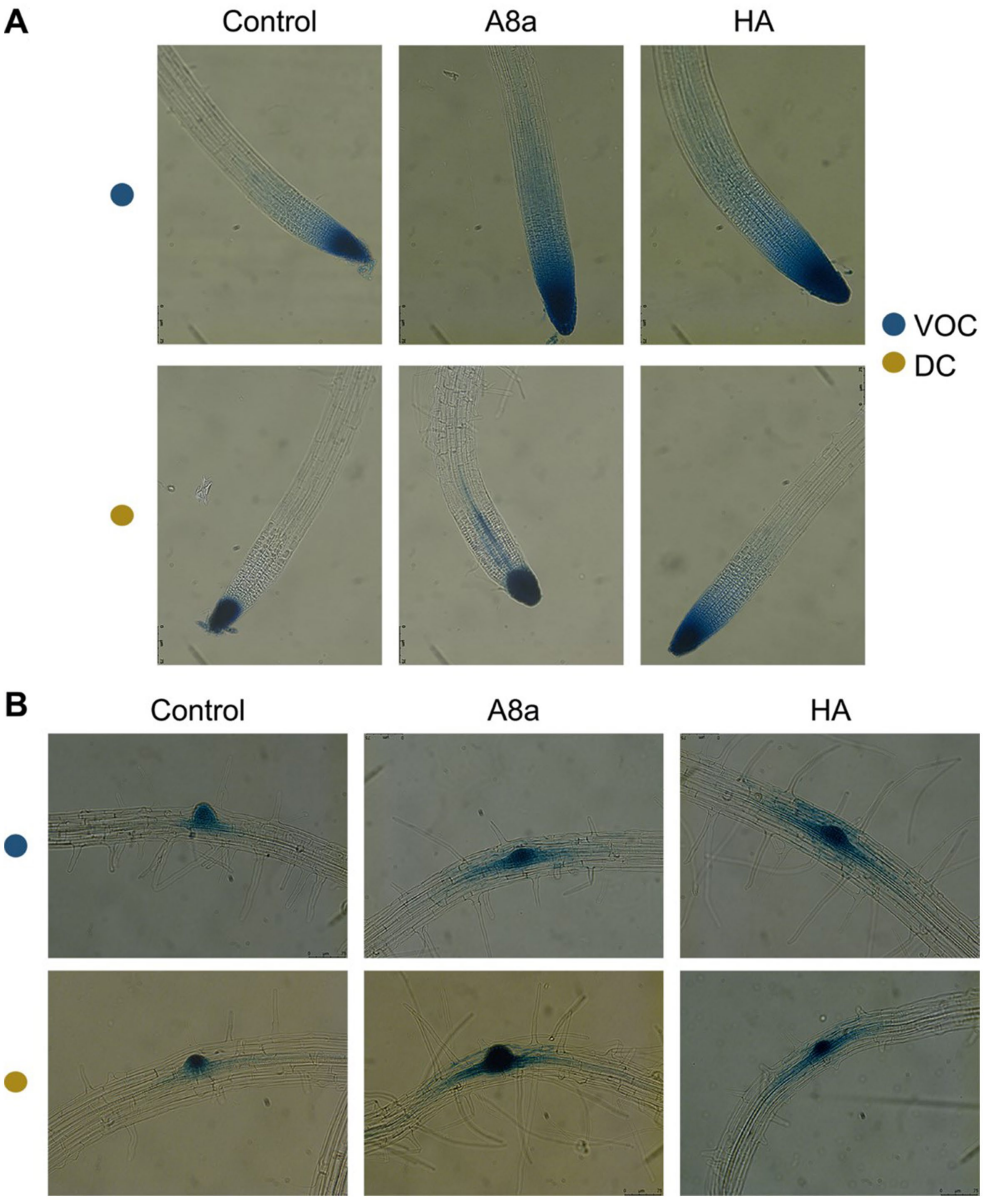
Effect of bacterial co-cultivation and their emitted VOCs on auxin-inducible gene expression in *Arabidopsis* primary and developing lateral roots. **(A)** GUS expression in primary root tips of *Arabidopsis DR5:GUS* seedlings. **(B)** *DR5:GUS* expression in the pericycle and emerging lateral roots. Photographs show representative images of at least 10 seedlings. Scale bars mean 150  μm.

To explore if root system remodeling induced by *Bacillus* spp. A8a and HA could correlate with an accumulation of endogenous auxin in *Arabidopsis* roots, we analyzed the expression of the responsive marker *DR5*:*GUS* in primary root tips, lateral root primordia and emerging lateral roots in transgenic *A. thaliana* seedlings co-cultivated with the bacterial strains. The exposure to VOCs from both strains visibly modified the area of accumulation of the marker, extending its expression through root tissues in an area up to three-fold greater than in the control and in developing primordia (Figure 4). Diffusible compounds from strain A8a caused the relocation of auxin, causing an enhanced auxin-inducible expression in the vasculature of primary roots and in lateral root primordia. On the other hand, diffusible compounds produced by strain HA induced a generalized expression of the marker in an area up to two-fold higher than in the uninoculated control seedlings (Figure 4).

**Figure 5:**
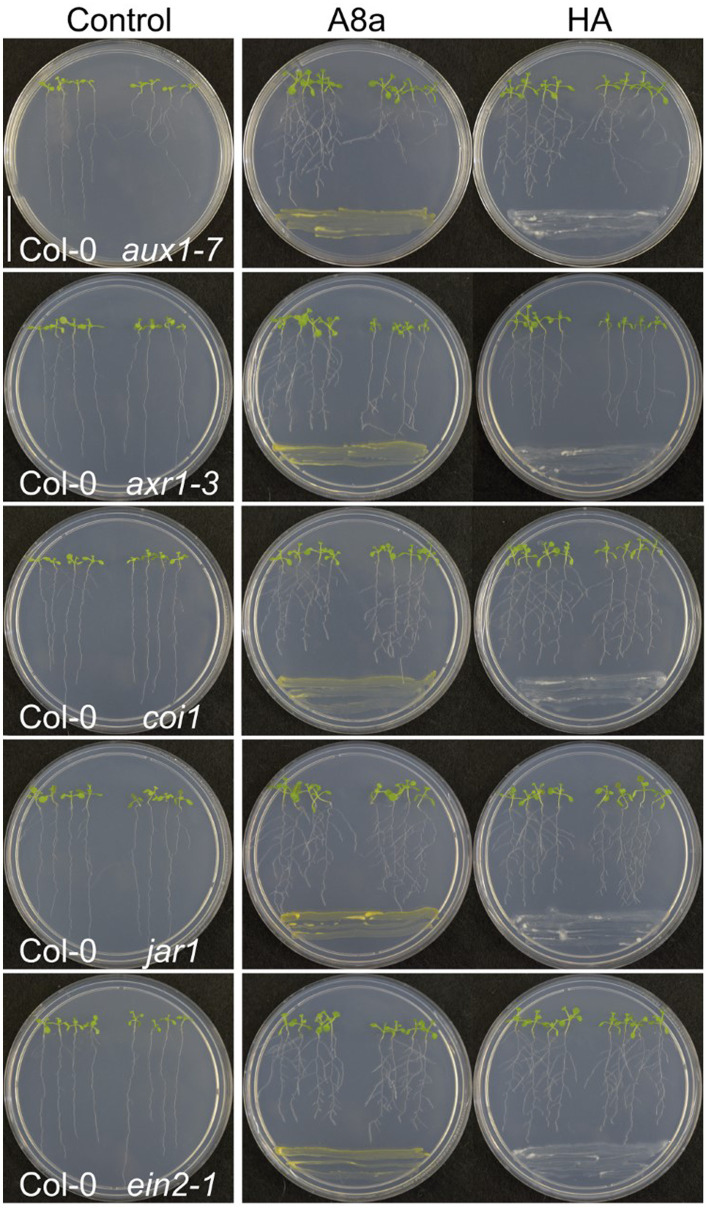
Effect of bacterial strains on root system architecture in *Arabidopsis* hormone-resistant mutants. Dual culture assays were established for 7  days with 7  day-old wild type (Col-0) or mutant seedlings. Photographs are representative from three replicates. Note the moderate increase in lateral root development in the auxin signaling-related mutant *axr1-3*.

To determine the involvement of auxin and other hormone-related signaling in developmental effects of bacteria co-cultivation, we performed a screening to analyze the possible resistance of *A. thaliana* mutants, affected in the auxin response (*aux1-7* and *axr1-3*), in JA perception (*coi1-1* and *jar1*) and in ethylene (ET) response (*ein2-1*). In terms of primary root inhibition, all tested mutants showed the same response than wild type *A. thaliana* ecotype Col-0 seedlings, however, the induction of lateral root formation in the auxin-related signaling mutant *axr1-3* was diminished compared to Col-0 (Figure 5). These results suggest that induction of lateral root formation by strains A8a and HA involves the auxin signaling pathway.

### 3.5. Defense responses induced by bacterial plant growth promoting diffusible or volatile compounds

**Figure 6:**
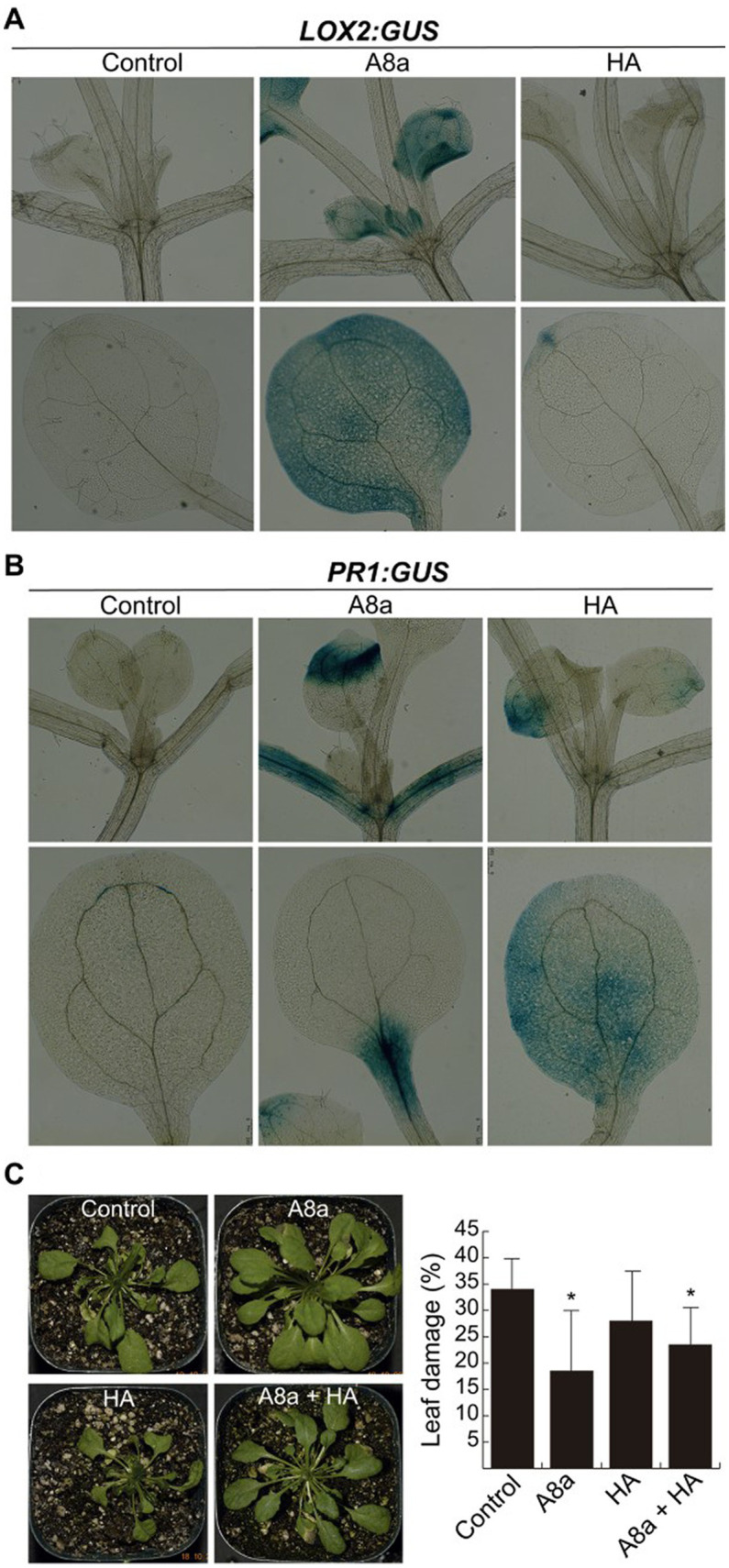
Induction of defense responses in *Arabidopsis* seedlings by bacterial diffusible or volatile compounds. **(A)** Effect of bacterial VOCs on the jasmonic acid-inducible gene expression *LOX2:GUS*. Seven day-old seedlings grown on MS-containing plates were inoculated at 2.5  cm from the root tip and co-cultured for 7  days and histochemically analyzed. **(B)** Expression of the salicylic acid-responsive gene *PR1:GUS* after 24 h of bacterial inoculation was histochemically analyzed. At least 10 seedlings per condition were recorded for the GUS expression assays. **(C)** Effect of long-term co-cultivation with bacterial strains in *Arabidopsis* plants, leaf-infected with *Fusarium solani*. At 16  days after fungal inoculation, plants were visually analyzed and the percentage of damaged leaf area was recorded. Values represent average ± standard deviation (*n* = 10–15); different letters indicate statistically significant differences compared with the mock-inoculated control (Tukey test, *p* ≤ 0.05).

A desirable trait searching for plant growth promoting bacteria is the ability to activate the plant defensive responses via JA and SA signaling. The effects of VOCs and diffusible compounds emitted by strains A8a and HA on defense-related gene expression of *A. thaliana* were evaluated by histochemically analyzing transgenic lines expressing the JA marker *LOX2:GUS* or the SA responsive gene *PR1:GUS*. An enhanced GUS expression was recorded in the leaves and petioles of seedlings exposed to VOCs emitted by *Bacillus* sp. A8a for the *LOX2* gene marker (Figure 6A), whilst VOCs emitted by strain HA did not induce the expression of *LOX2:GUS.* The *PR1:GUS* expression was induced by seedling exposition to both bacterial strains (Figure 6B).

To corroborate whether enhanced expression of defense genes activated the induced systemic resistance (ISR), *Arabidopsis* plants inoculated with rhizobacteria and growing in substrate were infected at the leaf level with *F. solani*. Single inoculation with strain A8a and the consortium A8a + HA significantly reduced the area of damage leaf up to 46 and 30%, respectively (Figure 6C).

## 4. Discussion

Rhizobacteria, in particular those from the *Bacillus* genus, constitute a promising source of specialized metabolites with antifungal/anti-oomycete activity and plant growth promotion properties (Aloo et al., 2019; Kiesewalter et al., 2021). Our findings contribute to elucidate the chemodiversity of two *Bacillus* strains isolated from the avocado rhizosphere and to further understand their bioactivity and the molecular mechanisms of their plant-microbe interactions. Using *A. thaliana* as a model non-natural host to study the influence of beneficial rhizobacteria on plant growth and development, hormonal homeostasis and ISR, we showed that short- and long-term bacterial treatments promoted root branching, shoot growth and biomass production.

The VOCs emitted by both bacterial strains were able to significantly reduce the growth of *F. kuroshium in vitro*, whilst only VOCs produced by *Bacillus* sp. A8a inhibited the growth of *P. cinnamomi*. The use of commercial standards allowed us to unambiguously identify some of the compounds from the volatile profiles of both bacterial strains; we thus corroborated their antagonistic activity against *Fusarium* spp., through *in vitro* growth inhibition assays and through the observation of hyphal damage by CSLM. The most effective compounds in reducing the growth of *Fusarium* spp. were 2,3,5-trimethylpyrazine, DMDS, 2-nonanone and 3-methyl-1-butanol. Although these compounds have been previously reported in volatile profiles of bacterial strains with antifungal activity, the confirmation of their fungicidal properties has been rarely provided. Exceptions include reports of antifungal activity of 2,3,5-trimethylpyrazine against *F. solani* (Guevara-Avendaño et al., 2019), of DMDS against *Sclerotinia minor*, *Sclerotinia sclerotium*, and *Verticillium dahliae* (Giorgio et al., 2015; Pecchia et al., 2017; Tyagi et al., 2020), of 2-nonanone against *S. sclerotium* (Giorgio et al., 2015), and of 3-methyl-1-butanol against *Botrytis cinerea* and *S. minor* (Calvo et al., 2020; Tyagi et al., 2020). Here, we report for the first time their effect against the emerging pathogen *F. kuroshium*. Underlying mechanisms of the fungicidal activity exhibited by these VOCs include cell wall disruption, alteration of membrane permeability, distortion of hyphae through vacuolization, and a reduction in ergosterol production through gene downregulation, inducing changes in fungal growth and pathogenicity (Giorgio et al., 2015; Tyagi et al., 2020; Weisskopf et al., 2021).

The VOCs emitted by the two selected bacterial strains were also able to influence plant growth and development by modifying root architecture and activating the ISR response, as shown by Fincheira and Quiroz (2018). In particular, the ability of VOCs emitted by strain HA to modify root growth in *A. thaliana* could be attributed to the presence of ketones and alcohols as predominant compounds in the volatile profile. Ketones such as acetoin, 2-nonanone and 2-heptanone, and alcohols such as 2,3-butanediol, were reported as dominant in the VOC profile of strain *B. amyloliquefaciens* L3, a PGPR which improved primary root growth, stimulated the formation of lateral roots and increased the total fresh weight of *A. thaliana* through VOC emission (Wu et al., 2019). Farag et al. (2006) also identified various alcohols as major compounds produced by two *Bacillus* strains that showed ability to induce *Arabidopsis* growth. The volatile 3-methyl-1-butanol, produced by some bacterial strains isolated from different agaves and cacti, some of them from the genus *Bacillus*, displayed beneficial effects for the growth and development of *A. thaliana*, *Agave tequilana*, and *Agave salmiana* (Camarena-Pozos et al., 2019). The identification of these compounds and that of some other esters and alcohols with previous bioactivity reports in the volatile profile of *Bacillus* spp. HA and A8a calls for future research to discern the individual and collective effects of pure VOCs on plant stimulation and fungal antagonism.

Our results also confirm that bacterial VOC emissions are temperature-dependent, VOC profiles being more diverse for both bacterial strains at 27°C and 30°C than at 23°C. These findings are in agreement with Misztal et al. (2018), who suggest that reduced bacterial metabolic growth rates and compound volatility at colder temperatures may be responsible for lower volatile emissions when culture temperature decreases. We show that, in the volatile profile of *Bacillus* sp. A8a, the relative abundance of 2,3,5-trimethylpyrazine increased at 23°C whilst that of DMDS decreased compared with the other tested temperatures. On the other hand, 3-methyl-1-butanol was only emitted by *Bacillus* sp. HA at 23°C. Characterizing volatiles profiles at temperatures lower than those required for optimum *in vitro* bacterial growth remains necessary to get a deeper understanding of bacterial VOCs emitted in the rhizosphere under natural conditions.

Bacterial diffusible compounds are another source of bacterial compounds with potential antifungal activity. Contrary to our previous studies where crude extracts were obtained from *Bacillus* spp. (Báez-Vallejo et al., 2020; Guevara-Avendaño et al., 2020), the results presented here showed that EtOAc extracts from both bacterial strains, applied at 2 mg ml^−1^, exhibited a stronger inhibition of *Fusarium* spp. and *P. cinnamomi* than n-BuOH extracts. Dimkić et al. (2017) also reported *Bacillus* EtOAc extracts to be the most efficient at antagonizing bacterial pathogens such as *Xanthomonas arboricola* and *Pseudomonas syringae*. Moreover, unlike most reports of *Bacillus* crude extracts with antifungal activity (Gong et al., 2015; Báez-Vallejo et al., 2020; Guevara-Avendaño et al., 2020; Jiao R. et al., 2021; Wu et al., 2021), we did not find lipopeptides among the tentatively identified compounds in the EtOAc crude extracts, in neither bacterial strain. This may be due to the lack of genes or of biosynthetic gene clusters in the strain genomes that are encoding for lipopeptides, which will be corroborated through future genomic analyses, or to the production of lipopeptides by both strains in quantities below the UPLC-QTOF-MS detection levels. Instead, we detected compounds such as polyketides, non-ribosomal peptides and the plant growth regulator IAA.

Macrolactins (detected in both strains), difficidin (detected in crude extracts from *Bacillus* sp. HA) and bacillaene (detected in crude extracts from *Bacillus* sp. A8a), are polyketides with reported antifungal properties (Caulier et al., 2019). Macrolactins have been hypothesized to be responsible for the antagonistic activity of diverse *Bacillus* strains against *F. graminearum* (Chen et al., 2018), *F. oxysporum* (Yuan et al., 2012), *Alternaria alternata* and *Rhizoctonia* sp. (Salazar et al., 2020). Difficidin is a less studied compound but has been shown to inhibit the growth of several bacterial plant pathogens, including *Erwinia amylovora*, *Ralstonia solanacearum*, and *Xanthomonas oryzae*, most likely through the damage of their cell wall and membrane and the impairment of their motility (Chen et al., 2009; Wu et al., 2015; Yuan et al., 2022). Additionally, bacillaene has also been suggested to be responsible for the antimicrobial activity of several *Bacillus* strains against *Corynespora cassiicola* (Xu et al., 2020), *Fusarium* sp. (Um et al., 2013) and a broad range of bacteria (Wu et al., 2021), mainly through the inhibition of protein synthesis (Patel et al., 1995). Genes involved in the biosynthesis of these polyketides have been identified in the genome of *B. amyloliquefaciens* subsp. *plantarum* strain B9601-Y2, a rhizobacterium with strong plant growth promoting activity in wheat (He et al., 2013).

Bacilysin, a non-ribosomal peptide tentatively identified in the EtOAc crude extracts from both *Bacillus* strains, has been reported for its antibacterial (Chen et al., 2009; Wu et al., 2015), antifungal (Caulier et al., 2018; Xu et al., 2020) and anti-oomycete activity (Han et al., 2021), mainly through the inhibition of chitin production and downregulation of genes related to macromolecule biosynthesis. The fact that bacilysin was produced by *Bacillus* sp. A8a and *Bacillus* sp. HA, combined with its broad range of action against prokaryotic and eukaryotic pathogens, makes it a promising compound for the biocontrol of *F. solani*, *F. kuroshium*, and *P. cinnamomi*. Future research should aim at isolating the pure compound from the bacterial crude extracts to confirm its antagonistic activity and evaluate its potential damage in hyphae.

Additionally, we detected the auxin IAA in EtOAc extracts obtained from both bacterial strains. Although IAA may not be directly involved in the antimicrobial activity displayed by *Bacillus* sp. A8a and *Bacillus* HA, it has been reported to modify root architecture and promote the growth of diverse plants species such as *A. thaliana* (López-Bucio et al., 2007), common duckweed (Idris et al., 2007), or rice (Liu et al., 2020). For example, Asari et al. (2017) reported that diffusible compounds produced by *B. amyloliquefaciens* UCMB5113 increased the extension of lateral roots and the elongation and formation of root hairs in *A. thaliana*, whilst inhibiting the growth of the primary root; strain UCMB5113 was further shown to produce IAA and cytokinins, which were most likely involved in the growth promoting ability displayed by the bacterium. The diffusible compounds produced by *Bacillus* spp. A8a and HA reconfigured the root system architecture of *A. thaliana* seedlings, which evidenced an auxin accumulation in the meristematic zone of primary roots and in developing lateral roots. These findings suggest that auxin relocation and signaling activation could modulate the beneficial effects of strains A8a and HA on plant development.

To elucidate the mechanisms behind rhizobacteria-mediated growth promotion and enhanced auxin response in the root system, the phytohormone-related *A. thaliana* mutants *aux1-7*, *axr1-3*, *coi1-1*, *jar1*, and *ein2-1* were co-cultivated with strains HA and A8a. The screening for resistance to primary root inhibition and root branching indicated that neither the auxin, ethylene, or jasmonic acid pathways were required in inducing primary root shortening, but suggested that the auxin signaling component AXR1 was involved in the stimulation of lateral root development. The mutant line *axr1-3* has previously provided evidence of the key role of auxin signaling in mediating root morphology response to IAA-producing *Azospirillum* and *Microbacterium* strains (Gilbert et al., 2022). The *Auxin Resistant-1* (*AXR1*) gene is highly conserved in plants and is involved in auxin signaling through the ubiquitylation of auxin repressors, acting downstream of the receptor TIR1 (transport inhibitor response 1) protein (Ruegger et al., 1998). The specific involvement of upstream components of the auxin signaling pathway in the plant morphological responses to rhizobacteria HA and A8a should be further explored. Moreover, a deeper understanding of the multiple signaling pathways involved in the plant growth-related and defense responses (i.e., auxin, JA/ET and SA) is required to ensure a successful application of these strains in the field. Our histochemical analysis allowed to confirm the expression of the JA and SA gene markers *LOX2:GUS* and *PR1:GUS*, respectively, in *A. thaliana* seedlings inoculated with both bacterial strains or exposed to their VOCs. Additionally, strain A8a significantly reduced leaf damage in pot-cultivated *Arabidopsis* plants infected with *F. solani*. Collectively, our results indicate that *Bacillus* spp. A8a and HA and their specialized diffusible and volatile metabolites exert an important plant stimulation effect, as biopesticides and biofertilizers in a non-natural host, which evidence the potential of these two rhizobacterial strains to be applied in avocado and some other important horticultural crops.

## Data availability statement

The original contributions presented in the study are included in the article/Supplementary material, further inquiries can be directed to the corresponding authors.

## Author contributions

EC-M, EG-A, and Alejandro M-B conducted the study and analyzed the data. Alfonso M-B, JG-A, and FR designed the study. Alejandro M-B, Alfonso M-B, and FR supervised the *Arabidopsis* experiments. JM-V, EG-S, AK-M, MR-V, JG-A, and FR supervised the study of bacterial compounds. EC-M, Alfonso M-B, JM-V, EG-A, JG-A, and FR wrote the manuscript. JG-A and FR provided the funding. All authors contributed to the article and approved the submitted version.

## Funding

This study was funded by project SEP-CONACyT Ciencia Básica 2017–2018 number A1-S-30794 (Programa Presupuestario F003) and by the National Fund for Scientific and Technological Development (Fondo Nacional de Desarrollo Científico y Tecnológico or FORDECYT) grant number 292399. EC-M received a fellowship from CONACyT to support her M.Sc. studies and a mobility grant from the Postgraduate Program of Instituto de Ecología, A.C. Alejandro M-B received a UNAM-DGAPA postdoctoral fellowship.

## Conflict of interest

The authors declare that the research was conducted in the absence of any commercial or financial relationships that could be construed as a potential conflict of interest.

## Publisher’s note

All claims expressed in this article are solely those of the authors and do not necessarily represent those of their affiliated organizations, or those of the publisher, the editors and the reviewers. Any product that may be evaluated in this article, or claim that may be made by its manufacturer, is not guaranteed or endorsed by the publisher.

## Footnotes

^1^ https://metlin.scripps.edu/landing_page.php?pgcontent=mainPage

^2^ http://ecmdb.ca/

^3^ http://www.chemspider.com/
