# Supplementary material for: Biocontrol and plant growth promoting traits of two avocado rhizobacteria are orchestrated by the emission of diffusible and volatile compounds
Source: Front Microbiol. 2023 May 3;14:1152597. doi: 10.3389/fmicb.2023.1152597 (PMC10189041; doi:10.3389/fmicb.2023.1152597)

Supplementary Material

Biocontrol and plant growth promoting traits of two avocado rhizobacteria are orchestrated by the emission of diffusible and volatile compounds

**Elvis M. Cortazar-Murillo, Alfonso Méndez-Bravo, Juan L. Monribot-Villanueva, Edith Garay-Serrano, Ana L. Kiel-Martínez, Mónica Ramírez-Vázquez, Edgar Guevara-Avendaño, Alejandro Méndez-Bravo, José A. Guerrero-Analco, Frédérique Reverchon**

*** Correspondence:**

José A. Guerrero-Analco
joseantonio.guerrero@inecol.mx

Frédérique Reverchon
frederique.reverchon@inecol.mx

**
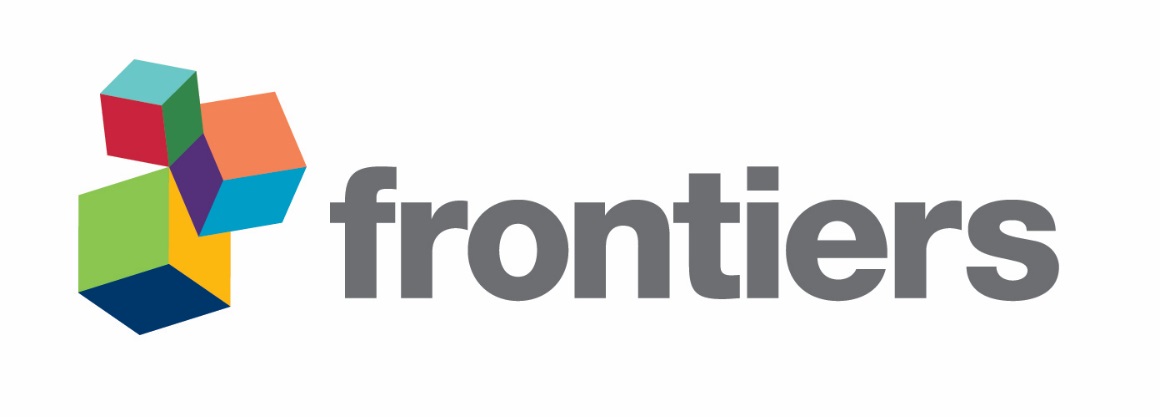
**

**Supplementary Material 1**. Percentages of inhibition of mycelial growth of the three tested pathogens by VOCs emitted by bacterial strains *Bacillus* sp. A8a and *Bacillus* sp. HA.

| **Pathogen** | **Percentages of inhibition (%)** | | | |
| --- | --- | --- | --- | --- |
|  | | 30 °C | 27 °C | 23 °C |
| *F. solani* | A8a | 23.61 ± 0.66 | 10.28 ± 0.55 | 3.29 ± 0.34 |
|  | HA | 26.77 ± 0.94 | 10.67 ± 0.79 | 4.93 ± 0.17 |
| *F. kuroshium* | A8a | N/A | 40.99 ±0.04* | 2.79 ±0.05 |
|  | HA | N/A | 27.28 ± 0.39* | 1.85 ± 0.13 |
| *P. cinnamomi* | A8a | 14.29 ± 0.49 | 76.00 ± 0.66^§^ | 2.96 ±0.03 |
|  | HA | 6.32 ±0.18 | 13.66 ± 0.20 | 0.83 ± 0.12 |

Values represent average ± standard deviation (n=3).

* Significantly different from the control (Kruskal-Wallis test, *P* ≤ 0.05)

§ Value reported in our previous study (Méndez-Bravo et al., 2018)

N/A: Not applicable, this incubation temperature was not available at the quarantine facility for this assay.

**Supplementary Material 2**. CSLM images of *F. solani* and *F. kuroshium* mycelium exposed to commercial VOCs (concentration: 3 M) for seven days. Yellow arrows highlight morphological distortions. Scale bar: 40 µm; objective plan achromatic, 63× (NA 1.4, immersion).


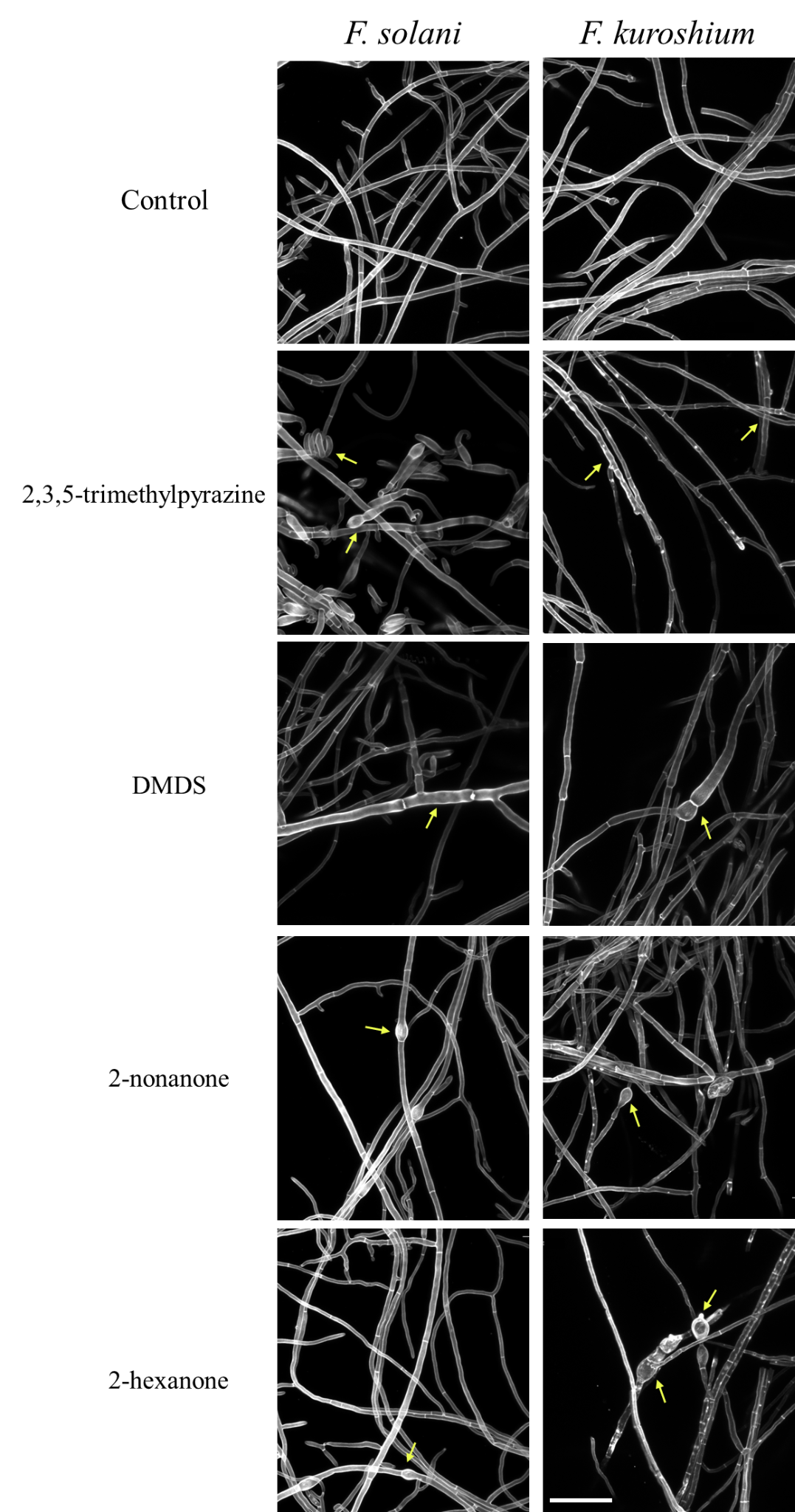


Supplementary Material 3. Percentages of inhibition of mycelial growth of the three tested pathogens in dual culture with *Bacillus sp*. A8a and *Bacillus sp*. HA.

| **Pathogen** | **Percentage of inhibition (%)** | |
| --- | --- | --- |
| *F. solani*  (30 °C) | A8a | 0.07 ± 0.09 |
|  | HA | 0.00 ± 0.10 |
| *F. kuroshium*  (27 °C) | A8a | 0.00 ± 0.16 |
|  | HA | 0.00 ± 0.25 |
| *P. cinnamomi*  (27 °C) | A8a | 19.66 ± 0.16* |
|  | HA | 2.98 ±0.04 |

Values indicate average ± standard deviation (n=3).

* significant differences compared with the control (mycelial growth without bacteria) (Dunn’s method, *P*≤0.05).

**Supplementary Material 4**. Mycelial growth inhibition by EtOAc crude extracts (2 mg mL^−1^) produced by *Bacillus* sp. A8a and *Bacillus* sp. HA against A) *F. solani*, B) *F. kuroshium*, and C) *P. cinnamomi*, after 72 h.


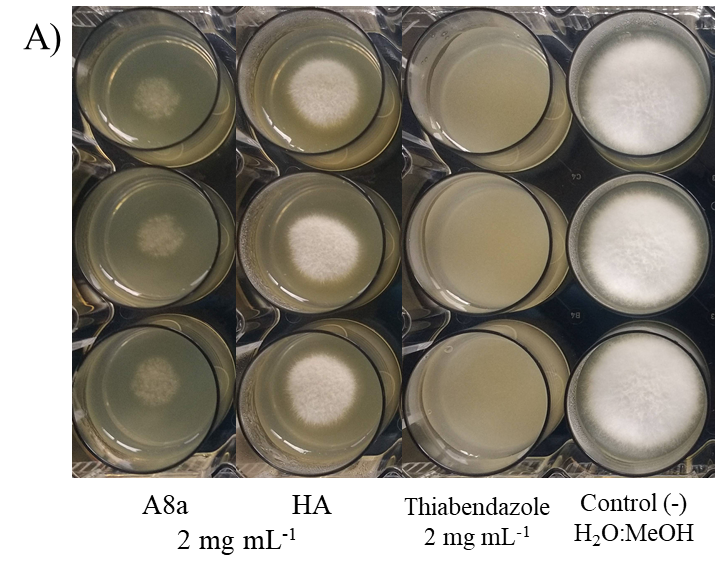

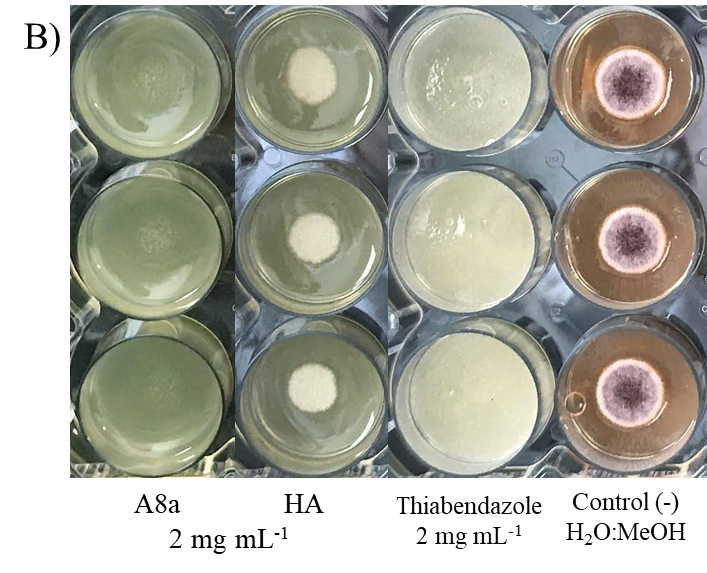


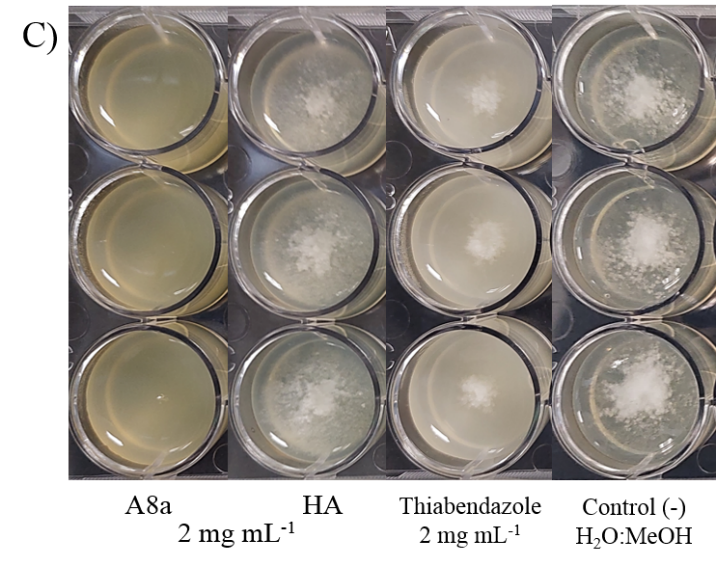


**Supplementary Material 5.** Long-term plant growth promotion activity of *Bacillus* sp. A8a, *Bacillus* sp. HA and their combination on *A. thaliana* growing in substrate. A) Number of leaves; B) aerial fresh weight; C) aerial dry weight.

Values represent average ± standard deviation (n=10 in plant growth evaluation assays, n=15 in biocontrol assays).

Different letters indicate significant differences (Tukey test, *P≤*0.05).


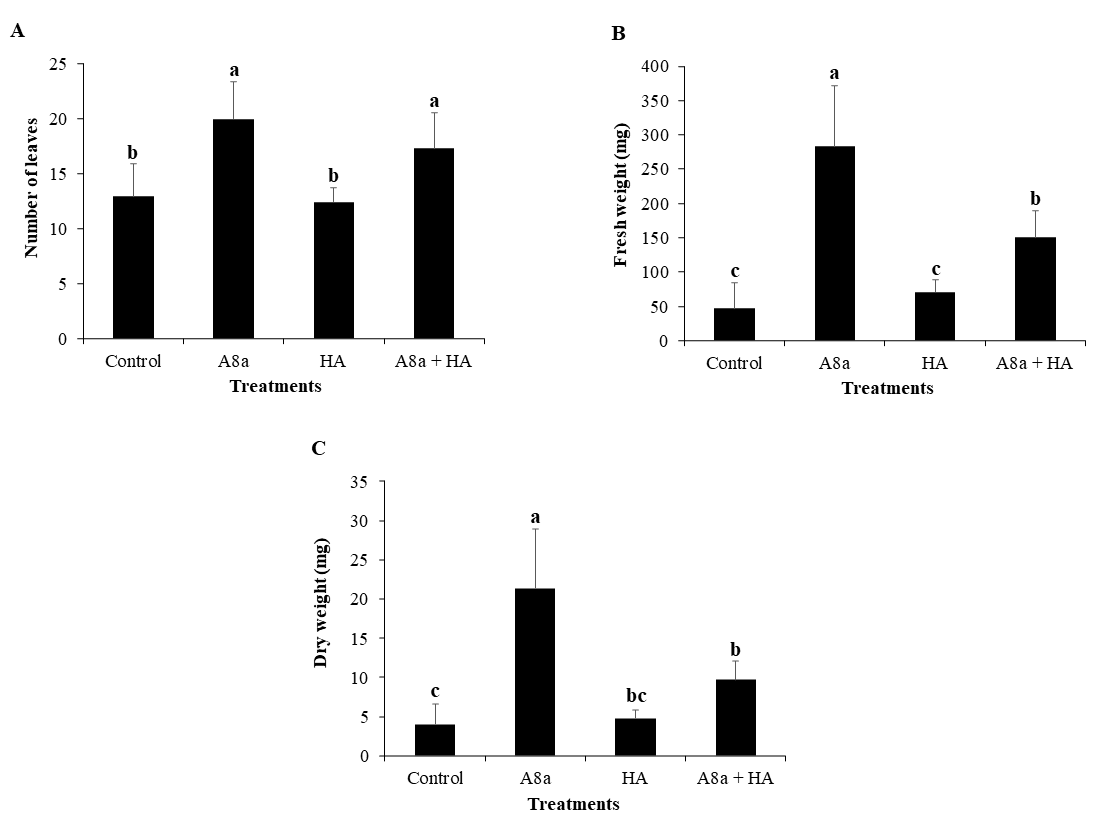

Supplement: Supplementary file 1 [file Data_Sheet_1.docx]
